# DNA hydroxymethylation controls cardiomyocyte gene expression in development and hypertrophy

**DOI:** 10.1038/ncomms12418

**Published:** 2016-08-04

**Authors:** Carolina M. Greco, Paolo Kunderfranco, Marcello Rubino, Veronica Larcher, Pierluigi Carullo, Achille Anselmo, Kerstin Kurz, Thomas Carell, Andrea Angius, Michael V. G. Latronico, Roberto Papait, Gianluigi Condorelli

**Affiliations:** 1 Humanitas Clinical and Research Center, Via Manzoni 56, Rozzano (MI) 20089, Italy; 2 Institute of Genetics and Biomedical Research, National Research Council of Italy, Via Manzoni 56, Rozzano (MI) 20089, Italy; 3 Center for Integrated Protein Science, Department of Chemistry, Ludwig-Maximilians-Universität München, 8137 Munich, Germany; 4 Humanitas University, Via Manzoni 56, Rozzano (MI) 20089, Italy; 5 Department of Cardiovascular Sciences, University of Leicester, Leicester LE3 9QP, UK

## Abstract

Methylation at 5-cytosine (5-mC) is a fundamental epigenetic DNA modification associated recently with cardiac disease. In contrast, the role of 5-hydroxymethylcytosine (5-hmC)—5-mC's oxidation product—in cardiac biology and disease is unknown. Here we assess the hydroxymethylome in embryonic, neonatal, adult and hypertrophic mouse cardiomyocytes, showing that dynamic modulation of hydroxymethylated DNA is associated with specific transcriptional networks during heart development and failure. DNA hydroxymethylation marks the body of highly expressed genes as well as distal regulatory regions with enhanced activity. Moreover, pathological hypertrophy is characterized by a shift towards a neonatal 5-hmC distribution pattern. We also show that the ten-eleven translocation 2 (TET2) enzyme regulates the expression of key cardiac genes, such as *Myh7*, through 5-hmC deposition on the gene body and at enhancers. Thus, we provide a genome-wide analysis of 5-hmC in the cardiomyocyte and suggest a role for this epigenetic modification in heart development and disease.

## Abstract (web summary)

5-hydroxymethylation of cysteine (5-hmC) plays a role in epigenetic regulation. Here the authors analyse the hydroxymethylome in embryonic, neonatal, adult and hypertrophic mouse cardiomyocytes and show that the dynamic modulation of hydroxymethylated DNA is important for cardiomyocyte gene expression programming in heart development and failure.

## Graphical abstract

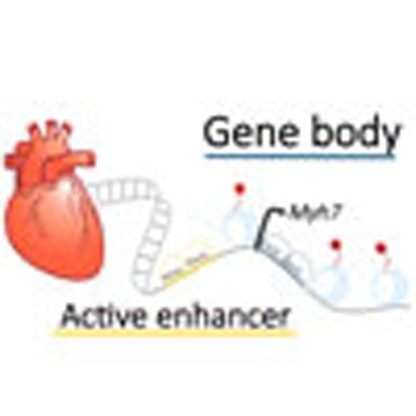

The heart can adapt to stimuli via the activation of specific transcriptional programmes. The epigenome—the sum of the chemical modifications occurring on DNA and the histone proteins it is wrapped around—has emerged as a key regulatory element of this process during heart development^1^ and disease^2^. Among the epigenetic modifications that make up the epigenome, methylation of DNA at position 5 of cytosine (5-mC) has been found to be involved in X-chromosome inactivation, transposon silencing and genomic imprinting^3^. Regarding the heart, whole-genome bisulfite sequencing of DNA methylation has recently revealed the plasticity of the 5-mC profile in the cardiac genome^4^.

DNA methylation was initially considered a stable epigenetic mark and until recently was the only known chemical modification of DNA. However, the discovery of 5-hydroxymethylcytosine (5-hmC)—an oxidation product of 5-mC catalysed by the ten-eleven translocation (TET) family proteins—has revised this notion^5^. In fact, studies have shown that 5-hmC is not only an intermediate product of an active demethylation process, but can also act as a relatively stable epigenetic mark whose role in transcription regulation is linked to its genomic location. Indeed, enrichment of 5-hmC on the gene body (GB) has been associated with the activation of transcription^6^,^7^,^8^,^9^,^10^,^11^. However, its function on promoters and distal regulatory regions is less clear. In embryonic stem cells (ESCs), for example, 5-hmC can localize to the transcription start site (TSS) of repressed, but developmentally poised, genes^7^,^12^,^13^; moreover, it can be highly enriched at poised and active enhancers^14^,^15^,^16^, but only recently have two studies proven that TET-mediated 5-mC oxidation regulates the activity of these elements^16^,^17^.

5-hmC has been investigated extensively in several cell and tissue types, but its relevance for myocardial pathophysiology is unknown. We therefore decided to profile the distribution of 5-hmC in the cardiomyocyte (CMC) genome. We found that DNA hydroxymethylation is highly dynamic during cardiac development and disease. Deposition of 5-hmC on the GB is strongly positively correlated with gene expression and marks cardiac-specific genes. Expression of the fetal cardiac α-myosin heavy chain (MHC) gene *Myh7* is tightly linked to both genic- and enhancer-associated hydroxymethylation: indeed, the locus undergoes extensive loss of 5-hmC on the GB during cardiac maturation and acquires the epigenetic mark on a distal regulatory region on the induction of hypertrophy. Moreover, 5-hmC deposition at, and expression of, *Myh7* in embryonic CMCs is profoundly affected by TET2 knockdown (KD).

## Results

### The 5-hmC landscape in development and hypertrophy

CMCs were isolated from mice at four experimental points: E14.5 embryos, 1- or 2-day-old pups, 2-month-old adult mice and adult mice subjected for 1 week to transverse aortic constriction (TAC), a surgical procedure that induces pathological cardiac hypertrophy due to pressure overload^18^. The purity of the cell populations was routinely 80–85%, as evaluated by staining for cardiac sarcomeric actinin (Supplementary Fig. 1a); quantitative reverse transcriptase–PCR analysis confirmed high expression of CMC markers and barely detectable expression of fibroblast-specific markers in the CMC fraction (Supplementary Fig. 1b).

**Figure 1:**
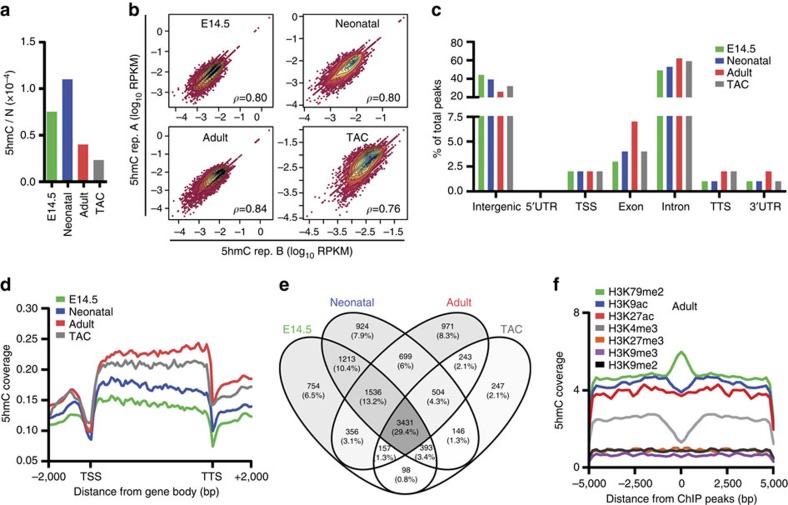
Genomic distribution of 5-hmC in mouse CMCs. (**a**) Quantification of global 5-hmC in embryonic (E14.5), neonatal, normal adult (Adult) and hypertrophic adult (TAC) CMCs. (**b**) Density plots illustrating the correlation of the GB 5-hmC level in biological replicates (rep. A and rep. B) of embryonic (E14.5), neonatal, normal adult and hypertrophic (TAC) CMCs. For each plot, Pearson's correlation coefficient *ρ* is given. Intragenic levels of 5-hmC were quantified by counting the number of reads falling onto genes from the TSS to the TTS. Read number was normalized to library size and gene length (RPKM). (**c**) The presence of the 5-hmC peaks overlapping in the biological replicates on different genomic elements. TSS, transcription start site; TTS, transcription termination site; UTR, untranslated region. (**d**) Average 5-hmC coverage across the GB of all reference genes (UCSC version mm10) at each studied point. (**e**) Venn diagram of the number of genes associated with the identified peaks at the four studied points; peaks overlapping with exons and introns were considered for the analysis. (**f**) Profiles of 5-hmC distribution around the H3K79me2, H3K9ac, H3K27ac, H3K4me3, H3K27me3, H3K9me3 and H3K9me2 peaks identified with MACS software in adult CMCs.

We first determined the absolute levels of 5-mC and 5-hmC in CMCs by isotope-based liquid chromatography–mass spectrometry^19^. The percentage of methylated DNA was up to fivefold lower in adult CMCs than in neonatal or embryonic cells (Supplementary Fig. 1c). A similar pattern was found for 5-hmC: hydroxymethylated DNA in adult CMCs was less than half of that in neonatal cells and was further lowered on the induction of hypertrophy (Fig. 1a).

Next, we examined the genomic distribution of 5-hmC by hydroxymethylated DNA immunoprecipitation (IP) coupled with high-throughput sequencing on two biological replicates per condition. We found 5-hmC-enriched sequences to result in more than 20 million reads in each condition. When mapped to the mouse genome (UCSC version mm10), these 5-hmC fragments generated more than ten million unique reads with a mapping quality >10 (Supplementary Fig. 1d). There was strong overlap between biological replicates regarding the genome-wide coverage profiles of raw unmapped reads (Supplementary Fig. 1e) and the intragenic level of 5-hmC mapped reads (Fig. 1b). 5-hmC density was similar across autosomes and the X chromosome, whereas the Y chromosome was largely depleted of 5-hmC, as previously observed in mouse ESCs^20^ (Supplementary Fig. 1f). Moreover, average 5-hmC content for both replicates correlated positively with the number of protein-coding genes on each chromosome at all experimental points (Supplementary Fig. 1g).

We then used HOMER software to identify the peaks of 5-hmC-enriched regions (Supplementary Dataset 1). To increase the significance of results, we used a highly stringent peak-selection criteria, selecting only peak base pairs overlapping in the biological replicates (Supplementary Fig. 1h). Of note, although global correlation of the biological replicates of the TAC condition was comparable to that in embryonic, neonatal and adult experiments, the percentage of overlapping peaks was lower, suggestive of higher variability and dynamicity of the pathological condition, a finding in line with the strong transcriptional reprogramming occurring in hypertrophic CMCs.

Most 5-hmC peaks were located on introns and intergenic regions (Fig. 1c), and the modification was always less present at TSSs and transcription termination sites (TTSs) (Fig. 1d). Specifically, 5-hmC was more enriched at intergenic regions in fetal CMCs, was distributed mainly (69%) on the GB (introns plus exons) in adult CMCs and hypertrophy was characterized by a re-distribution to intergenic regions, a profile similar to that of embryonic and neonatal CMCs. Relative enrichment of 5-hmC on GBs was highest in adult CMCs (Fig. 1c) and did not reflect the changes observed in absolute genomic 5-hmC quantification (Fig. 1a), as previously observed in the hippocampus^9^.

A total of 11,672 protein-coding genes were enriched for 5-hmC during development and hypertrophy and ∼70% underwent re-distribution of 5-hmC deposition (Fig. 1e). Integrative analyses of histone modifications indicated that dimethylation of lysine 79 on histone H3 (H3K79me2)—a modification distributed preferentially to the GB and recently implicated in CMC differentiation^21^—strongly co-localized with 5-hmC, whereas trimethylation of lysine 4 (H3K4me3)—a mark mainly enriched around the TSS—had an opposite trend (Fig. 1f).

Thus, during cardiac development, 5-hmC is lost by intergenic regions and tends to gradually accumulate on the GB; moreover, hypertrophy is characterized by a shift towards a neonatal-like distribution pattern. The latter point is significant considering that the pathologically hypertrophic heart is characterized by suppression of the adult gene programme and reversion to a more neonatal-like one^22^.

To investigate whether the ‘immature' 5-hmC landscape of hypertrophic CMCs was biologically meaningful, we hierarchically clustered genic hydroxymethylation enrichment. This revealed a subset of 528 genes in hypertrophic CMCs having a 5-hmC distribution pattern similar to that in neonatal cardiac cells (Supplementary Fig. 2a,b). These genes were highly enriched for Gene Ontology (GO) terms such as regulation of heart rate, heart development and fatty acid oxidation (FAO), pathways known to undergo ‘neonatal-like' reprogramming in hypertrophic CMCs (Supplementary Fig. 2c,d).

### Genic 5-hmC correlates with gene expression

**Figure 2:**
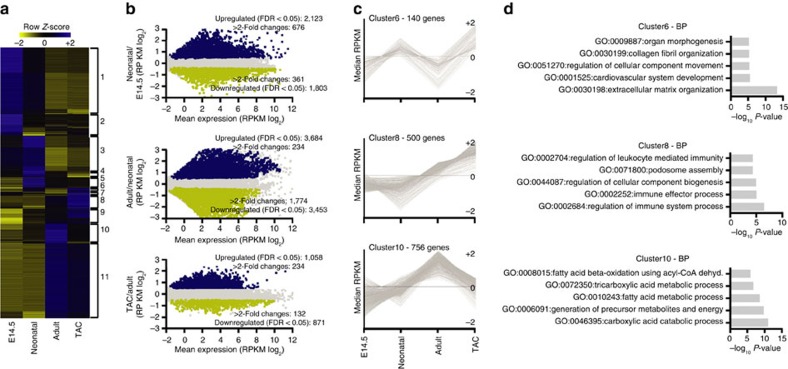
Transcriptional analysis during development and hypertrophy. (**a**) Hierarchical clustering of the 10,302 transcripts expressed differentially among embryonic, neonatal, normal adult and hypertrophic CMCs (adjusted *P*-value ≤0.05, Benjamini–Hochberg correction for multiple-testing). SOTA cluster numbers are indicated to the right. (**b**) Comparison of gene expression in the developmental and pathological transitions by RNA-seq analysis. Shown are summary dot plots, with blue dots representing upregulated genes and yellow dots representing downregulated genes (*n*=2 samples each; adjusted *q*-value ≤0.05). (**c**) Examples of SOTA clustering analysis of the 10,302 differentially expressed genes. (**d**) Enriched GO terms of the relative SOTA cluster. GO analysis was performed with Metascape 1.0 (*P*-value ≤0.01).

We performed deep RNA sequencing (RNA-seq) to assess the relationship between 5-hmC and gene expression (Supplementary Fig. 3a). Overall, there were >11,500 expressed protein transcripts (reads per kilobase of transcript per million reads mapped (RPKM) ⩾1). Of these, pairwise differential analysis identified 10,302 that were differentially expressed among the four conditions (Fig. 2a,b and Supplementary Dataset 2). These differentially modulated genes were grouped by SOTA software into 11 clusters with distinct patterns of expression (Fig. 2c and Supplementary Fig. 3b) and encoding for unique biological features (Fig. 2d and Supplementary Fig. 3c).

**Figure 3:**
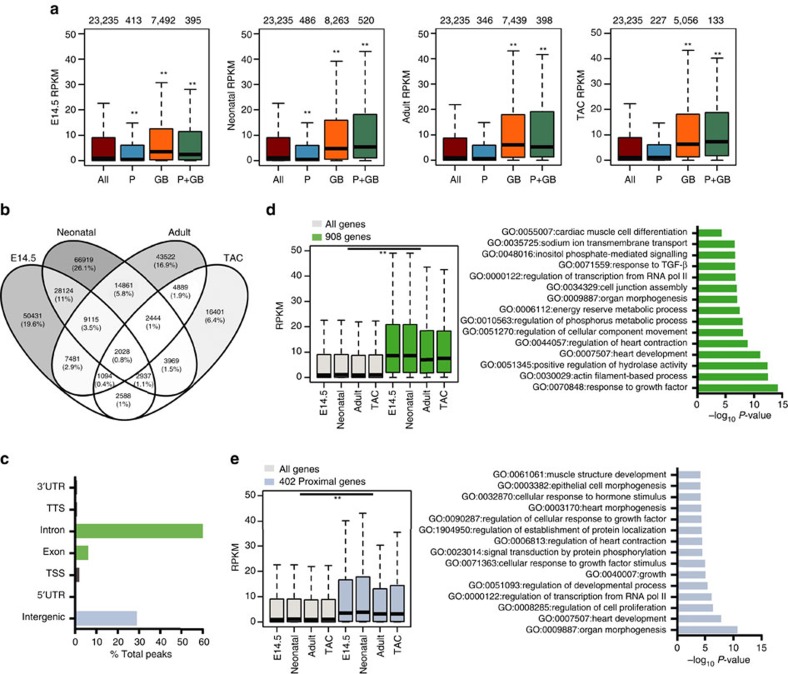
Correlation of 5-hydroxymethylcytosine and gene expression. (**a**) Boxplots of RNA expression (RPKM) of all genes (brown bars), genes with 5-hmC exclusively present at promoters (P, blue bars), genes with 5-hmC exclusively on the GB (orange bars) and genes with 5-hmC at the promoter and on the GB (P+GB, green bars). Thick lines indicate the median, with whiskers extending to ±1.5 of the interquartile range. The number of genes in each group is shown above the plots. **P-value ≤0.01 (Mann–Whitney test). RPKM, reads per kilobase per million mapped reads. Promoters were defined as −1 kb to +1 kb relative to the TSS. (**b**) Venn diagram showing the number of common 5hmC-enriched intervals (peaks) across the four studied points, identified with bedtools multinter tool. (**c**) The presence of the 5-hmC peaks overlapping all four studied points (*n*=2,028) at different genomic elements. TSS, transcription start site; TTS, transcription termination site; UTR, untranslated region. (**d**) Boxplots of RNA expression (RPKM) of the 908 genes with at least one peak on the GB (exons plus introns) at all studied points (left). Thick lines indicate the median, with whiskers extending to ±1.5 of the interquartile range. **P-value ≤0.01 (Mann–Whitney test). RPKM, reads per kilobase per million mapped reads. Enriched GO terms relative to the 908 genes (right). GO analysis was assessed with Metascape 1.0 (*P*-value ≤0.01). (**e**) Boxplots of RNA expression (RPKM) of the 402 genes with at least one peak overlapping all the studied points proximal to the TSS (intergenic) (left). Thick lines indicate the median, with whiskers extending to ±1.5 of the interquartile range. **P-value ≤0.01 (Mann–Whitney test). RPKM, reads per kilobase per million mapped reads. Enriched GO terms relative to the 402 genes (right). GO analysis was assessed with Metascape 1.0 (*P*-value ≤0.01).

Genes with 5-hmC on their GB were significantly more expressed than the median of all genes (Fig. 3a). In neonatal, adult and hypertrophic cells, median 5-hmC GB coverage increased progressively along the expression quartiles, a relationship not evidently present in embryonic CMCs (Supplementary Fig. 4a). The effect of GB 5-hmC on gene expression was cumulative: the deeper the intragenic coverage, the greater the gene expression (Supplementary Fig. 4b).

In contrast, exclusive enrichment of the modification at promoters was associated with mild repression of gene expression in embryonic and neonatal CMCs, and no effect on adult (non-proliferating) CMCs (Fig. 3a). When present on the promoter and on the GB, 5-hmC maintained a positive correlation with gene expression, indicating that the effect on the GB is predominant. These findings are suggestive of different transcriptional roles for 5-hmC in embryonic and postmitotic CMCs, and indicate that the effect of 5-hmC on gene expression is position dependent.

Given its broad distribution in the cardiac genome and the strong association of intragenic 5-hmC with gene expression, we hypothesized that the modification marks constitutively active genes. We thus identified and studied the common 5-hmC-enriched peak intervals of the four conditions (Fig. 3b). As expected, they were mainly distributed to intronic and intergenic regions (Fig. 3c). Genes hydroxymethylated on the GB at all experimental points were significantly more expressed (Fig. 3d); these genes encoded proteins for fundamental cellular processes, such as regulation of transcription and actin-filament-based process, as well as for processes specific to the cardiac cell, such as regulation of heart contraction. Likewise, common intergenic hydroxymethylated regions were found proximal to 402 genes that were also significantly more expressed (Fig. 3e); these were enriched for terms such as regulation of transcription, signal transduction and heart development. This suggested a possible role for 5-hmC also on distal regulatory regions. Thus, 5-hmC is associated in the CMC with highly expressed genes involved in ubiquitary and cell-specific functions, as has been reported for other cell types^6^,^8^,^9^,^11^.

Coherently, there were clear positive associations between 5-hmC on the GB and the presence of active histone marks that we have previously studied in adult and hypertrophic CMCs (specifically, H3K79me2, H3K9ac, H3K27ac and H3K4me3)^23^; we found no significant relationships with the repressive histone marks H3K27me3, H3K9me3 or H3K9me2 (Supplementary Fig. 5a,b). In the absence of an active mark, GB 5-hmC was only mildly associated with increased expression; in contrast, the presence of 5-hmC with an active histone mark at the GB was strongly associated with enhanced gene expression (Supplementary Fig. 5c).

### Changes of 5-hmC on repeated sequences

Repetitive DNA elements have been shown to be differentially methylated in the heart of cardiomyopathic patients^24^. As 5-hmC was redistributed to intergenic regions in hypertrophic CMCs, we examined its presence on these repeated sequences. Almost all classes of DNA repeats were generally enriched for 5-hmC (Supplementary Fig. 6a). Moreover, these elements are known to undergo transcription in various tissues^25^,^26^. In line with this, there was a strong positive correlation between 5-hmC coverage of repeated sequences and their expression in CMCs (Supplementary Fig. 6b).

The overall coverage of 5-hmC on repetitive elements became markedly reduced during cardiac development, but was partially restored with the induction of hypertrophy (Supplementary Fig. 6c), especially on long interspersed nuclear elements (LINEs) (Supplementary Fig. 6d). Of note, the enrichment of 5-hmC at LINEs was specific to hypertrophic CMCs (Supplementary Fig. 6e). Moreover, the histone modification H3K9me3—a key indicator of LINE repression^27^—was significantly depleted on hydroxymethylated LINEs in hypertrophic CMCs (Supplementary Fig. 6f). Enrichment of 5-hmC over these elements in hypertrophic CMCs was confirmed by glucosyl tagging^28^ (Supplementary Fig. 6g).

To assess whether hydroxymethylation of LINE-1 was associated with demethylation of CGs in hypertrophic CMCs, we performed TET-assisted bisulfite sequencing (TAB-seq) coupled with regular bisulfite sequencing (Supplementary Fig. 6h). The analysed DNA region underwent strong hydroxymethylation accompanied by a pronounced loss of CG methylation. Thus, in the hypertrophic heart 5-hmC may have a role at LINES in rendering chromatin structure more permissive.

### Differential 5-hmC correlates with specific gene programmes

**Figure 4:**
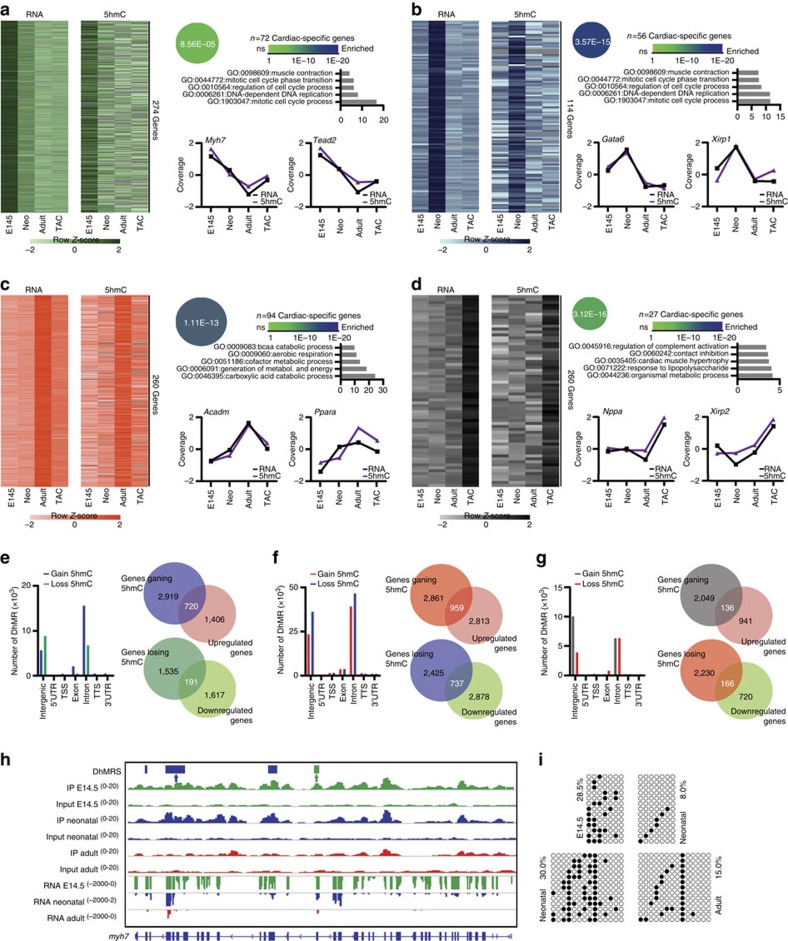
Dynamic and highly correlated 5-hmC and gene expression patterns during differentiation. (**a**–**d**) Heat map of log_2_-transformed 5-hmC enrichment values and gene expression values for the embryonic (**a**), neonatal (**b**), adult (**c**) and hypertrophic (**d**) clusters. Beside each heat map are: enrichment of genes within the cardiovascular expression set of the Mouse Genome Informatics (MGI) database (www.inforamatics.jax.org), calculated with Fisher's exact test, enriched GO terms and example genes. 5-hmC and gene expression are given as median-normalized values. (**e**–**g**) Association of all identified DhMRs within different genomic elements in the embryonic-to-neonatal (**e**), neonatal-to-adult (**f**) and adult-to-TAC (**g**) transitions (left). Venn diagrams show the overlaps between the number of genes undergoing solely 5-hmC acquisition on the GB (exons plus introns) and upregulation of gene expression (assessed by RNA-seq) (right top), and the number of genes undergoing solely 5-hmC loss on the GB and downregulation of gene expression (right bottom). (**h**) IGV profile of 5-hmC MeDIP-seq and RNA-seq profiles of the cardiac-specific gene *Myh7*. DhMRs identified with MeDIPS are indicated by boxes above the tracks (green, DhMRs in the embryonic-to-neonatal transition; blue, DhMRs in the neonatal-to-adult transition). Scales for each track are shown to the left. Exact locations of primers used to validate DhMRs are shown above the IGV profile (green arrow, embryonic-to-neonatal transition; blue arrow, neonatal-to-adult transition). Primer sequences are listed in Supplementary Table 2. (**i**) Validation of 5-hmC by single-base resolution analysis (TAB sequencing) in embryonic-to-neonatal (top) and neonatal-to-adult (bottom) transitions. Black circles indicate hydroxymethylated CpGs; open circles indicate unmethylated CpGs.

We then examined how DNA hydroxymethylation and gene expression were coordinated during cardiac maturation and hypertrophy. Four stage-specific clusters were computed, each one containing genes with comparable GB hydroxymethylation and expression patterns (Fig. 4a–d). All clusters were significantly enriched for cardiac genes: cluster 1 (Fig. 4a) contained genes with peaks in hydroxymethylation and expression at the embryonic stage, and included *Myh7*; cluster 2 (Fig. 4b) contained genes with peaks at the neonatal stage and included heart development genes such as *Gata6*; cluster 3 (Fig. 4c) contained key genes for adult heart metabolism, such as *Ppara*; and cluster 4 (Fig. 4d) contained genes with the highest expression and genic hydroxymethylation during hypertrophy, such as the cardiomyopathy-associated gene *Xirp2*.

Next, we performed differential enrichment analysis to identify differentially hydroxymethylated regions (DhMRs) (Supplementary Dataset 3). Overall, genes acquiring 5-hmC on the GB had increased expression, whereas genes losing 5-hmC on the GB had decreased expression (Supplementary Fig. 7a). Specifically, there was a significant (*P*<0.01, Mann–Whitney test) association between gain of 5-hmC and induction of gene expression in the embryonic-to-neonatal and neonatal-to-adult transitions; in contrast, there was no globally significant correlation on induction of hypertrophy, even though DhMRs were found on a specific set of significantly modulated genes in this setting.

The embryonic-to-neonatal transition was characterized by a predominant gain of 5-hmC within intragenic elements (Fig. 4e). 5-hmC was acquired on 720 significantly upregulated genes involved mainly in energy production and metabolism (Supplementary Fig. 7b); among these were 270 cardiac-specific genes (Supplementary Fig. 7c). Loss of 5-hmC was instead associated with downregulation of 191 genes (54 of which were cardiac specific) that were enriched for biological processes related to tissue morphogenesis, embryonic development and Wnt signalling (Supplementary Fig. 7a,c).

Further gain of 5-hmC on intragenic elements occurred in the neonatal-to-adult transition (Fig. 4f): these DhMRs were found on 959 upregulated genes (193 of which were cardiac specific; Supplementary Fig. 7d) enriched for energetic and metabolic pathways (Supplementary Fig. 7b). Loss of 5-hmC was associated with the downregulation of 737 genes (including 276 cardiac-specific ones) involved in heart development, angiogenesis and cell adhesion (Supplementary Fig. 7a,d).

In contrast, hypertrophy was associated with pronounced loss of 5-hmC on intragenic elements and a marked gain on intergenic regions (Fig. 4g): despite the large overall loss of intragenic hydroxymethylated DNA in hypertrophic CMCs, the modification was gained by a set of 40 upregulated heart-specific genes (Supplementary Fig. 7e) among a total of 136 genes involved mainly in extracellular matrix organization and actin cytoskeleton organization (Supplementary Fig. 7b), two important processes for the development of hypertrophy^29^. Finally, loss of 5-hmC was associated with repression in a total of 166 genes (56 of which were cardiac-specific) belonging to processes related to the tricarboxylic acid (TCA) cycle, fatty acid metabolism and the generation of energy, a finding consistent with the impaired energy production of hypertrophic CMCs^30^(Supplementary Fig. 7a,e).

Several DhMRs identified in the three transitions were selected for validation by two independent methodologies: TAB-seq^14^ and biotin-glucosyl tagging and enrichment^28^. They confirmed differential hydroxymethylation at the analysed loci in an independent cohort of samples (Supplementary Fig. 8a–e). Of note, *Myh7* underwent extensive loss of DNA hydroxymethylation at the GB during cardiac maturation (Fig. 4h,i), thereby indicating that 5-hmC may be a novel determinant of the regulation of the fetal isoform of cardiac MHC.

In the mammalian fetal brain, 5-hmC pre-marks certain genomic regions, facilitating their active demethylation at later stages of development^31^. To investigate whether hydroxymethylation contributes to CG demethylation also during cardiac development, we took advantage of a previously generated DNA methylome data set^4^. We found that genes undergoing a loss of mCG and/or hmCG in adult versus neonatal CMCs in Hein's data set were strongly hydroxymethylated at the neonatal stage. Moreover, 5-hmC abundance correlated with the level of demethylation: the greater the degree of developmentally driven CG demethylation of genes, the greater the hydroxymethylation at the demethylated genes in neonatal CMCs (Supplementary Fig. 7f).

Therefore, 5-hmC follows a specific distribution pattern during CMC maturation and disease, contributing to the specification of transcriptional programmes tightly linked to the biology of these cardiac cells.

### 5-hmC defines a subset of enhancers with increased activity

Enhancers are key regulators of tissue-specific gene expression programmes. Sets of distal enhancer regions have been recently reported for cardiac differentiation^32^ and hypertrophy^23^. Moreover, studies on ESCs have found high levels of 5-hmC at enhancers^15^,^33^. Therefore, we assessed the presence of 5-hmC on these regulatory regions in cardiac cells. We found strong associations between 5-hmC and regions classified as active (H3K27ac^+^/H3K4me1^+/−^) or poised (H3K4me1^+^ only) enhancers by Bruneau's group in CMCs differentiated *in vitro* from ESCs^32^. Consistent with findings in ESCs^34^, 5-hmC was depleted at the centre of active, but not of poised, enhancers (Supplementary Fig. 9a). Moreover, genes proximal to a 5-hmC^+^ enhancer were expressed at higher levels than those near a 5-hmC^−^ one (Supplementary Fig. 9b).

**Figure 5:**
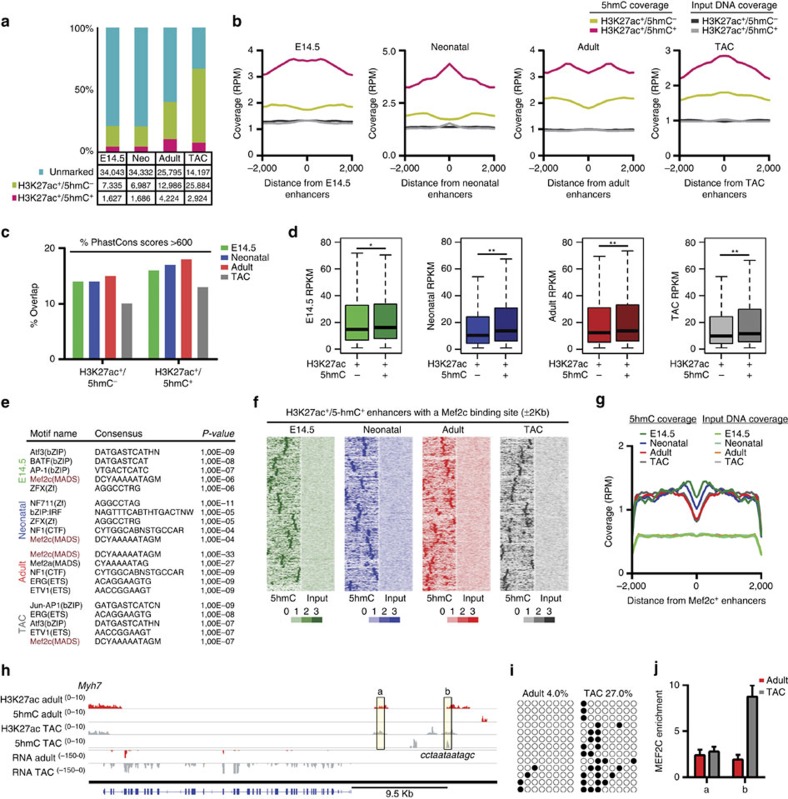
5-hmC-enriched enhancers. (**a**) Total enhancers identified in embryonic (E14.5), neonatal (Neo), adult and hypertrophic CMCs (TAC), categorized by H3K27ac and 5-hmC status. (**b**) Profiles of 5-hmC and input DNA coverage on H3K27ac^+^ enhancers in embryonic, neonatal, adult and hypertrophic CMCs. RPM, read counts per million mapped reads. Distance from enhancers in bp. (**c**) H3K27ac^+^/5-hmC^+^ enhancers display increased overlap with highly conserved placental mammal PhastCons scores (>600) than do H3K27ac^+^/5-hmC^−^ enhancers. (**d**) RNA-seq expression values in embryonic, neonatal, adult and hypertrophic CMCs for the single nearest gene associated with a H3K27ac^+^/5-hmC^−^ or H3K27ac^+^/5-hmC^+^ enhancer. Boxplots give the median (bold line), with whiskers extending to ±1.5 of the interquartile range. *P-value ≤0.05; **P-value ≤0.01 (Mann–Whitney test). (**e**) Top five binding-site motifs identified on H3K27ac^+^/5-hmC^+^ enhancers in embryonic, neonatal, adult and hypertrophic CMCs. Enriched motifs analysis on genomic regions was performed with HOMER findMotifsGenome. (**f**) Heat maps of 5-hmC and input DNA densities across the H3K27ac^+^/5-hmC^+^ enhancers containing a Mef2c binding-site motif. (**g**) Average profiles of 5-hmC and input DNA coverage across the Mef2c binding-site motif identified on H3K27ac^+^/5-hmC^+^ enhancers. RPM, read counts per million mapped reads. Distance from enhancers in bp. (**h**) IGV profile of H3K27ac-enriched regions, 5-hmC-enriched regions and RNA-seq profiles in normal adult and hypertrophic (TAC) CMCs, indicating an H3K27ac^+^/5-hmC^+^ enhancer region located upstream of *Myh7*. The predicted binding site for Mef2c and the consensus sequence are shown. Genomic regions validated through ChIP assays are indicated by the boxed regions (**a**,**b**). Scales for each track are shown to the left. (**i**) Validation of 5-hmC by single-base resolution analysis (TAB sequencing) of the H3K27ac^+^/5-hmC^+^ enhancer region located upstream of *Myh7*. Black circles indicate hydroxymethylated CpGs; open circles indicate unmethylated CpGs. The exact location of primers used to validate selected regions are listed in Supplementary Table 2. (**j**) ChIP analysis of the binding of Mef2c in normal adult (red bars) and hypertrophic (grey bars) CMCs in two regions: (**a**) control region; (**b**) Mef2c motif-positive region. Data are presented as mean±s.d. (*n*=3).

To dissect the functional role of enhancer hydroxymethylation, we performed chromatin IP sequencing (ChIP-seq) for H3K27ac. Only peaks common to two replicates were considered for analysis (Supplementary Fig. 9c). Peaks not overlapping any exon or promoter region (±1 kb from the TSS) were considered putative enhancers (Supplementary Fig. 9d). A total of 43,005 putative H3K27ac^+^ enhancer regions were identified at the four points studied (Supplementary Dataset 4). Of these, a subset was specifically enriched for 5-hmC (Fig. 5a,b). Only 94 H3K27ac^+^/5-hmC^+^ enhancers were preserved across all stages, whereas most were stage specific, in line with the notion that enhancers are highly cell-type specific^32^.

Enhancer hydroxymethylation may be important for the specification of transcriptional programmes in CMCs. In fact, both preserved and non-preserved 5-hmC^+^ enhancers were located in the proximity of transcription factor genes known to play pivotal roles in cardiac cells (that is, *Mef2a*, *Mef2c* and *Gata4*) as well as key cardiac genes (for example, *Myh7)* (Supplementary Fig. 9e). Enhancer conservation analysis identified an increased overlap of highly conserved phastCons mammalian elements with hydroxymethylated enhancers versus non-hydroxymethylated enhancers at all four stages, suggesting that these 5-hmC-marked enhancer regions are more likely to be conserved (Fig. 5c). To test whether H3K27ac^+^/5-hmC^+^ enhancers correlated with specific gene expression patterns, we assigned each enhancer to a neighbouring gene. Notably, genes in close proximity to a 5-hmC-marked enhancer were statistically more expressed than those proximal to an enhancer marked only by H3K27ac (Fig. 5d). In line with the idea that hydroxymethylated enhancers are critical regulators in cardiac cells, GO analysis revealed an enrichment of heart development genes in all four stage-specific sets of hydroxymethylated enhancers (Supplementary Fig. 9f).

Consistently, motif discovery analysis identified within these regions a significant, common over-representation of binding sites for myocyte enhancer factor Mef2c—the primary member of the MEF family of transcription factors involved in transcriptional regulation of the developing and hypertrophic heart^35^ (Fig. 5e–g). Figure 5h illustrates an H3K27ac^+^/5-hmC^+^ enhancer with a putative binding site for Mef2c in the proximity of *Myh7*, which becomes strongly induced in the hypertrophic heart. TAB-seq and ChIP analysis confirmed the enrichment of 5-hmC and the binding of Mef2c to this genomic region (Fig. 5i,j).

### TET2 KD alters the 5-hmC profile and gene expression

**Figure 6:**
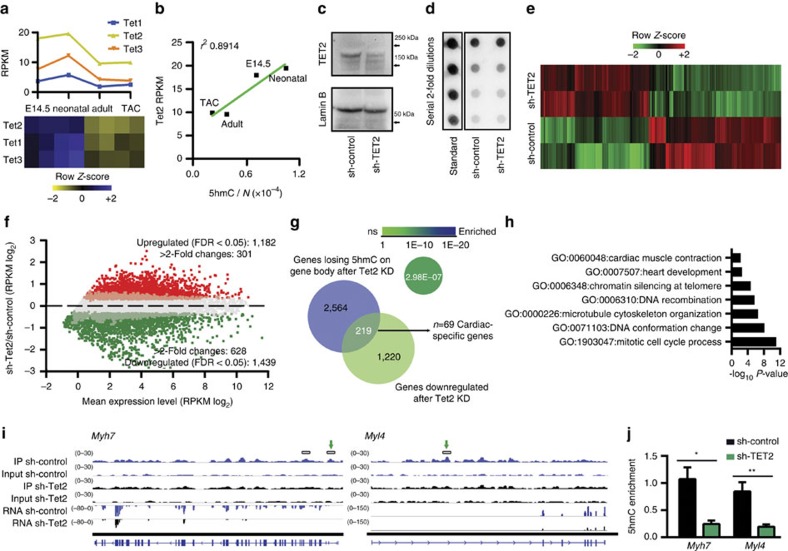
Involvement of TET2 in cardiac hydroxymethylcytosine deposition. (**a**) Transcript abundance during cardiac maturation and hypertrophy for the three TET family genes, assessed by RNA-seq. (**b**) Plot showing the positive correlation between the absolute content of 5-hmC (5-hmC/N), assessed by liquid chromatography–mass spectrometry and *Tet2* RNA expression (RPKM). (**c**) Expression of TET2 protein in CMCs expressing sh-Tet2 or sh-control. Embryonic CMCs were transduced with a lentiviral vector expressing a previously validated sh-Tet2 or a scrambled sequence (sh-control) and harvest 72 h after transduction. Lamin B was used to assess loading. Representative blot shown. Full-length blot is presented in Supplementary Fig. 10. (**d**) Dot blot analysis of genomic 5-hmC after KD of *Tet2*. (**e**) Heat map of hierarchical clustering of 1,182 upregulated and 1,439 downregulated genes after *Tet2* RNA KD, measured by RNA-seq on two biological replicates for each condition. (**f**) Comparison of gene expression (RNA-seq) in CMCs expressing sh-control or sh-Tet2. Shown is a summary dot plot, with red dots representing upregulated genes and green dots representing downregulated genes (*n*=2 samples each; *q*-value ≤0.05). (**g**) Venn diagram showing the overlap in the number of genes undergoing 5-hmC loss exclusively on the GB (exons plus introns) and downregulation of expression (RNA-seq) after *Tet2* KD. Enrichment of genes within the MGI cardiovascular expression database, calculated using Fisher's exact test. (**h**) Enriched GO terms relative to the 219 genes in panel (**g**). GO analysis was conducted with Metascape 1.0 (*P*-value ≤0.01). (**i**) IGV profiles of 5-hmC MeDIP-seq and RNA-seq profiles of representative genes (*Myh7* and *Myl4*). DhMRs identified with MeDIPS are shown by boxes above the profiles. The scale for each track is given to the left. Exact locations of primers used to validate DhMRs are shown by green arrows. (**j**) Analysis of 5-hmC enrichment on *Myh7* and *Myl4*. Data are mean±s.d. (*n*=3). *P-value ≤0.05; **P-value ≤0.01 (unpaired Student's *t*-test).

We next characterized the expression of TET family members in cardiac cells. RNA-seq data revealed that all three TET genes were highly expressed in fetal CMCs, became downregulated in adult cells and were not significantly modulated further on hypertrophy induction; the most highly expressed member was *Tet2* (Fig. 6a). Notably, absolute 5-hmC content correlated positively with *Tet2* expression (Fig. 6b). These findings led us to hypothesize that TET2 plays a role in fetal CMCs. We therefore examined the effect of *Tet2* KD by transducing cultured embryonic CMCs with a lentiviral vector expressing a previously validated short hairpin RNA targeting *Tet2* (sh-Tet2) or a scrambled sequence (sh-control)^36^ and conducted RNA-seq 72 h later (Supplementary Fig. 10a). sh-Tet2 significantly decreased endogenous *Tet2* expression (Supplementary Dataset 5); western blotting confirmed a 70% reduction in TET2 protein (Fig. 6c and Supplementary Fig. 11a). However, *Tet2* KD did not affect the global 5-hmC level (Fig. 6d and Supplementary Fig. 11b), a result attributable to compensation by *Tet3*, which was significantly upregulated on silencing of *Tet2* (Supplementary Dataset 5), as already observed in other primary cell types^37^. Nonetheless, *Tet2* disruption resulted in significant deregulation of a large number of genes (Fig. 6e,f): among differentially expressed genes were those related to cell cycle, heart development and heart muscle contraction (Supplementary Fig. 10b).

Next, to identify genes directly affected by *Tet2* KD, we mapped 5-hmC genomewide (Supplementary Fig. 10c). Biological replicates showed a high degree of genome-wide correlation (Supplementary Fig. 10d) and, consistent with the dot blot, *Tet2* KD did not affect global levels of 5-hmC on all genes (Supplementary Fig. 10e), suggesting it may affect 5-hmC at specific loci. We identified 159,365 regions of differential hydroxymethylation (Supplementary Dataset 6), which were mainly found in intronic and intergenic regions (Supplementary Fig. 10f). Thus, *Tet2* KD seemed to affect gene expression by targeting genic and distal regulatory regions.

Intragenic loss of 5-hmC was associated with a slight decrease in median expression, but it significantly affected a number of highly expressed genes (Fig. 6g and Supplementary Fig. 10g,h). Among the TET2**-regulated genes were many with pivotal functions for embryonic cardiac cells: cardiac muscle contraction and cardiac muscle fibre development were among the top-enriched terms, as were mitotic cell cycle processes (Fig. 6h). Notably, *Myh7* and the myosin light-chain gene *Myl4*—which encode essential structural components of embryonic cardiac muscle—were profoundly affected by*Tet2* KD, undergoing six- and threefold downregulation, respectively (Fig. 6i). The specific depletion of 5-hmC at intragenic regions of *Myh7* and *Myl4* was further confirmed by quantitative PCR (qPCR) of differential peaks (Fig. 6j).

**Figure 7:**
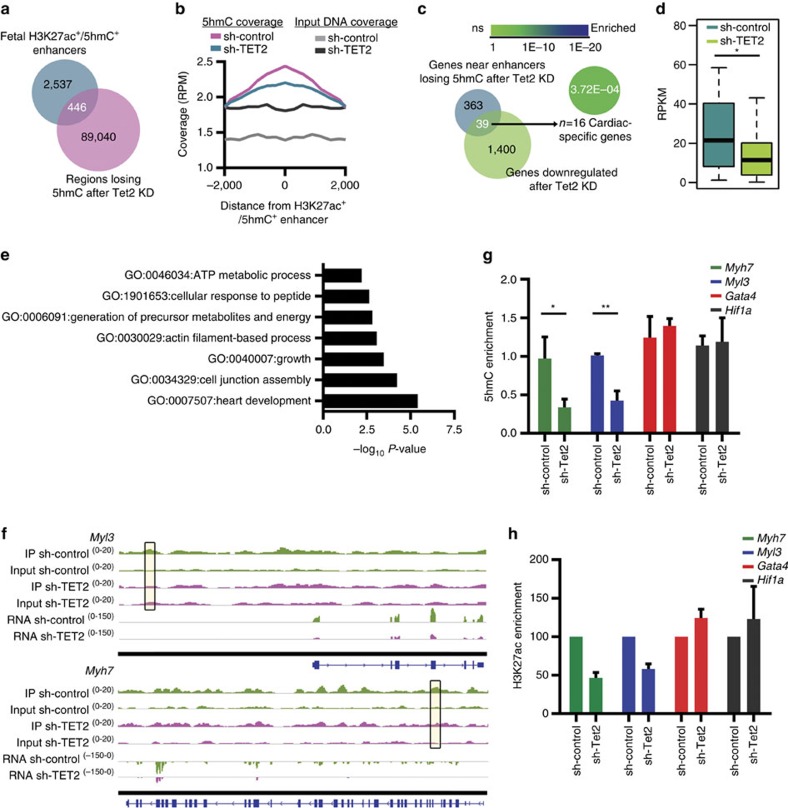
5-hmC-enriched enhancers affected by KD of TET2 enzyme. (**a**) Venn diagram showing the overlap in the number of H3K27ac^+^/5-hmC^+^ enhancers identified in fetal stages (E14.5 and neonatal CMC s) and the number of regions undergoing loss of 5-hmC after *Tet2* KD, identified by MeDIPS. (**b**) Average coverage profile of 5-hmC and input DNA coverage across the H3K27ac^+^/5-hmC^+^ enhancers undergoing loss of 5-hmC after *Tet2* KD. Distance from enhancer (bp) is given on the *x* axis. RPM, read counts per million mapped reads. (**c**) Venn diagram showing the overlap in the number of genes (*n*=402) proximal to the 446 H3K27ac^+^/5-hmC^+^ enhancers undergoing loss of 5-hmC and the number of genes downregulated (RNA-seq) after *Tet2* KD. Enrichment of genes within the MGI cardiovascular expression database, calculated with Fisher's exact test. (**d**) RNA-seq expression values in sh-Tet2 and sh-control CMCs for the single nearest gene associated with an H3K27ac^+^/5-hmC^+^ enhancer depleted of 5-hmC after *Tet2* RNA KD. Boxplots give the median (bold line), with whiskers extending to ±1.5 of the interquartile range. *P-value ≤0.01 (Mann–Whitney test). (**e**) Enriched GO terms relative to the 39 genes in **d**. GO analysis was performed with Metascape 1.0 (*P*-value ≤0.01). (**f**) IGV profile of 5-hmC MeDIP-seq and RNA-seq in sh-control and sh-Tet2 CMCs showing one intergenic (top) and one intragenic (bottom) H3K27ac^+^/5-hmC^+^ enhancer region depleted of 5-hmC (boxes) after *Tet2* RNA KD, respectively, proximal to *Myl3* and *Myh7*. Scale for each track is shown on the left. (**g**) qPCR analysis of 5-hmC enrichment on *Myl3*, *Myh7*, *Gata4* and *Hif1a* H3K27ac^+^/5-hmC^+^ enhancers. Data are presented as mean±s.d. (*n*=3). *P-value ≤0.05; **P-value ≤0.01 (unpaired Student's *t*-test). (**h**) ChIP analysis of the enrichment of H3K27ac on *Myl3*, *Myh7*, *Gata4* and *Hif1a* H3K27ac^+^/5-hmC^+^ enhancers. Data are presented as mean±s.d. (*n*=2).

Next, we assessed whether transcriptional perturbation in *Tet2* KD CMCs was also linked with altered hydroxymethylation on enhancers. We thus investigated variations in 5-hmC densities over the putative enhancer regions we previously identified as hydroxymethylated in fetal cells. A subset of these (*n*=446) underwent significant loss of 5-hmC following *Tet2* KD (Fig. 7a,b). This affected 39 genes (Fig. 7c), which became significantly repressed (Fig. 7d). Again, many genes involved in heart development and contraction (Fig. 7e), including *Myh7* and *Myl3* (Fig. 7f), were found in the proximity of a deregulated enhancer.

Given the association between activating histone marks and DNA hydroxymethylation, we asked whether 5-hmC acted as a pre-activating mark defining regions of strong transcriptional activation. We took advantage of *Tet2* KD to partially test this hypothesis. qPCR analysis of two enhancer regions that lost 5-hmC after *Tet2* KD (namely, proximal to *Myh7* and *Myl3*), indicated that depletion of 5-hmC led to decreased enrichment of H3K27ac at these regions (Fig. 7g,h and Supplementary Dataset 6); in contrast, two enhancers for which the level of 5-hmC deposition was not altered by *Tet2* KD (namely, proximal to *Gata4* and *Hif1a*), did not undergo any change in H3K27ac enrichment at the analysed time point. These findings identify TET2 and its catalytic product, 5-hmC, as new factors in the shaping of the epigenetic and transcriptional landscapes of maturing CMCs.

## Discussion

The present study reveals that DNA hydroxymethylation is critical for the gene expression programme of cardiac cells. We define the genome-wide map of hydroxymethylated DNA during cardiac development and disease, showing that 5-hmC marks gene bodies and distal regulatory regions in CMCs. We further show a functional link between TET2, DNA hydroxymethylation at the GB and enhancer, and regulation of gene expression in fetal cardiac cells. Together, these findings give new insights into mechanisms governing cardiac gene expression.

A previous investigation of the methylome of embryonic, postnatal and failing CMCs carried out with whole-genome bisulfite sequencing identified a large number of differentially methylated regions mainly on gene bodies and *cis*-regulatory sequences and highlighted a previously unappreciated plasticity of cardiac DNA methylation^4^. However, even though bisulfite sequencing allows resolution at the base level, it does not discriminate between 5-mC and 5-hmC^38^. We therefore asked whether 5-hmC could be an additional element of the CMC's methylome. In fact, the high abundance of 5-hmC in the central nervous system^39^,^40^ has encouraged several investigations into its function during neurogenesis^41^, postnatal neuronal development and ageing^9^,^31^,^42^: in those contexts, 5-hmC was found to be a stable, but dynamic, epigenetic mark. Moreover, quantification of 5-hmC in mammalian tissues suggested that heart and skeletal muscle contain intermediate levels of hydroxymethylated DNA^43^,^44^. Our findings reveal that 5-hmC marks active genes and is associated with distinct transcriptional networks during cardiac maturation—that is, genes involved in embryonic and heart development during the E14.5→neonatal transition and genes involved in energetic and metabolic pathways during the neonatal→adult transition—as well as in the development of cardiac hypertrophy. This suggests that dynamic modulation of hydroxymethylated DNA at gene bodies is a feature of the gene expression programme of CMCs. In addition, 5-hmC is enriched in neonatal CMCs at CG regions that become demethylated later in development, implicating this mark in the shaping of the methylome of adult heart, similar to what has been reported for the brain^31^.

A common response of the adult heart to various stresses is the suppression of the postnatal gene programme and the re-activation of a neonatal-like one. On one hand, fetal cardiac genes, such as those encoding atrial and brain natriuretic factor, are upregulated, as is the fetal contractile protein isoform of MHC MYH7 (ref. 45); on the other, the hypertrophied heart returns to a fetal metabolic gene profile by downregulating adult transcripts, such as those encoding mitochondrial FAO, respiratory chain complexes and TCA cycle enzymes, as well as genes involved in calcium handling^22^,^46^. Our findings reveal that the hydroxymethylome of TAC CMCs recapitulates this characteristic feature of the hypertrophied heart. In fact, we found that the 5-hmC landscape of hypertrophic CMCs is shifted, in part, towards a neonatal-like distribution pattern. More precisely, these CMCs display a predominant loss of 5-hmC within genic regions, a change mainly affecting genes related to TCA cycle, FAO and the generation of energy. In addition, gain of 5-hmC at intergenic regions is associated with re-activation of fetal genes such as *Myh7*, which we found to be in the proximity of an enhancer that was hydroxymethylated specifically in hypertrophic CMCs.

The sole presence of 5-hmC on the GB is not, however, correlated with high gene expression levels: it must be co-expressed with an activating histone mark, such as H3K79me2, H3K9ac, H3K27ac or H3K4me3, potentiating the effect of the latter. Thus, 5-hmC may be a pre-activating mark, attracting or precluding the binding of chromatin remodelling proteins.

The exact mechanism through which TETs and 5-hmC regulate transcription remains elusive. TET2 and TET3 have been shown to directly interact with *O*-linked β-*N*-acetylglucosamine transferase mediating histone O-GlcNAcylation^47^ and to influence the deposition of H3K4me3, which in turn promotes transcription of target genes^48^. Moreover, mass spectrometry-based analysis has identified a large set of tissue-specific proteins selectively bound to or repelled by hydroxymethylated DNA^49^. Here we show that depletion of 5-hmC significantly affects enrichment of the activating histone mark H3K27ac within enhancer regions. Whether this is due to a reduced binding of acetyltransferases (that is, CREB and p300) or by simply creating a more accessible chromatin structure remains to be elucidated.

TET2-mediated hydroxymethylation has also been associated with the modulation of enhancer activity in ESCs^16^,^17^. We show here that enhancer-associated 5-hmC plays a role in the regulation of transcription in cardiac cells: in fact, 5-hmC defined a subset of enhancers with increased activity in CMCs. Of note, the top biological function of genes in the proximity of hydroxymethylated enhancers was ‘Heart Development' and included several key cardiac regulators. Moreover, transcription network analysis revealed that the activity of cardiac-specific enhancers is regulated by MEF2C. Another transcription factor, GATA-4, has already been reported to shape the chromatin environment during cardiac development and disease and, in turn, regulate enhancer activity^42^. Similarly, MEF2C may be involved in establishing the epigenetic signature, in particular the deposition of 5-hmC on enhancers by binding with TETs, as recently observed for the transcription factor WT1 in HEK293T cells^50^.

We also determined that TET2 is the most abundantly expressed member of this enzyme family in CMCs, and that it is implicated in defining the hydroxymethylation profile of fetal cells. Even though *Tet2* KD did not affect global levels of hydroxymethylation, it led to an alteration of 5-hmC at specific loci, indicating that TET2-mediated hydroxymethylation regulates gene expression in a specific, rather than a broad, manner. Our findings provide the first evidence of a regulatory function of TET enzymes in cardiac cells. More specifically, inhibition of *Tet2* expression affects intragenic- and enhancer-associated hydroxymethylation, downregulating specific cardiac genes. Importantly, 5-hmC critically regulates the gene encoding MYH7. In fact, expression of *Myh7* appears to be driven by both genic and enhancer hydroxymethylation: the locus undergoes extensive loss of 5-hmC at the GB during cardiac maturation, but acquires this epigenetic mark on a distal regulatory region in hypertrophy. Indeed, there was a clear link between TET2 downregulation and alteration of 5-hmC deposition at these specific genic structures, changes associated with strong deregulation of *Myh7* expression. Further studies aimed at elucidating the specific role of TET2 and DNA hydroxymethylation in this context, possibly with inducible and tissue-specific models, are thus warranted.

The findings of the present study set the basis for the understanding of the functional role of 5-hmC in cardiac physiology and disease, opening the field to the investigation of novel mechanisms governing cardiac transcription.

## Methods

### Animal procedures

All experiments were performed according to the 2010/63/EU Directive and approved by the ethics committee of Humanitas Research Hospital. Mice were housed in a controlled environment on a 12 h light/dark illumination schedule and fed on a chow diet. The study was performed using genomic material obtained from CMCs isolated from embryonic (E14.5), neonatal (1–2 days old) and adult (2-month-old) male mice, and from adult male mice 1 week after TAC.

TAC surgery was adapted from Rockman *et al*.^51^. Briefly, C57Bl6/J male mice were anaesthetised by intraperitoneal injection of a mixture ketamine (100 mg kg^−1^) and xylazine (10 mg kg^−1^). The aortic arch was exposed through the first intercostal space. A 8-0 suture was passed between the truncus anonymous and the left carotid artery, and a knot tightened around a blunted 27-gauge needle (TAC group). The chest cavity was then closed with 6-0 silk sutures. A separate group of mice underwent the same surgical procedure but without any tightening of the knot (sham group). The pressure load caused by the knot was verified through measurement of the pressure gradient across the aortic constriction with echocardiography (Vevo 2100, VisualSonics).

Male mice were killed according to the protocol of the internal ethics committee, the hearts excised and CMCs isolated. Adult CMCs were obtained by enzymatic perfusion of the left ventricle with Liberaze TM (Roche), using a Langerdorff apparatus as described elsewhere^52^. Cells were then allowed to sediment by gravity in order to obtain separation of viable, rod-shaped CMCs from rounded, dead CMCs and from other cell types present in the heart.

E14.5 and neonatal CMCs were obtained by enzymatic digestion of excised ventricles^52^. Single cell suspensions were obtained by performing eight rounds of enzymatic digestion with 240 units per ml of type II collagenase (Worthington). The cell suspensions were then centrifuged at a low speed to allow initial separation of CMCs from other cell types. The supernatant was removed, the cells resuspended in 10 ml of isolation medium and then seeded in 10-cm culture dishes and incubated at 37 C for 1 h, to allow adhesion of non-myocytes. This step was repeated twice to minimize contamination from other cell types.

We routinely assessed the purity of our preparations by fluorescence-activated cell sorting (FACS) analysis and obtained 80–85% sarcomeric actinin-positive cells. As an additional control of the purity of the preparations used for 5-hmC DNA IP (hMeDIP), we verified the absence of 5-hmC peaks in intragenic regions of abundantly expressed endothelial and fibroblast markers (for example, *Vim*, *Col1a2* and *Tcf21*) (Supplementary Dataset 1).

### hMeDIP and sequencing

Two biological replicates were profiled at each experimental point. For each replicate, DNA was isolated using the QIAamp DNA mini kit (Qiagen). DNA was sonicated to ∼150–400 bp (distribution peak, ∼250 bp), Illumina adaptors ligated to the ends and immunoprecipitated with a 5-hmC antibody (Active Motif, AM39791). The IP reaction was performed with Active Motif's reagent kit and protocol.

After IP, DNA fragments were amplified with barcoded Illumina primers to generate the final sequencing library. To generate the input libraries, sonicated DNA with ligated adaptors was directly amplified without the IP step. High-throughput sequencing was carried out by Active Motif's epigenetic service (http://www.activemotif.com/catalog/821/MeDIP-sequencing-service) using the Illumina HiSeq 2000 platform.

### 5-hmC data processing and analysis

Raw sequencing reads were mapped to the UCSC mouse genome mm10 build using BWA 0.7.4-r385 (ref. 53) with the default parameter setting. Multiple mapped reads were filtered out using the MarkDuplicates module of the Picard command-line tools (http://broadinstitute.github.io/picard/). Reads with ‘mapQ' <10 were filtered out using bamtools API 2.2.3 tool.

Quality control and saturation analysis was assessed after read mapping with the MEDIPS R/Bioconductor package^54^. Saturation analysis indicated that our data set was sufficient to generate a saturated and reproducible coverage profile of the reference genome. Moreover, we computed effectiveness of hMeDIP enrichment by identifying the fraction of CpGs in the reference genome covered by the sequencing data and evaluating their depth of coverage.

Detection of 5-hmC-enriched regions relative to input DNA was carried out with the ‘findPeaks' routine of HOMER v4.5 in ‘histone' mode and with default parameter values. Results of HOMER analysis are included in Supplementary Data set 1. Only peaks identified in both biological replicates were considered for further analysis.

Analysis of DhMRs was carried out with the MEDIPS R/Bioconductor package^54^. Results of MEDIPS analysis are included in Supplementary Data sets 3 and 5. Significant DhMRs were chosen based on an edgeR *P*-value ≤0.05.

Intragenic levels of 5-hmC were quantified by counting the number of reads falling into UCSC genes defined by the corresponding annotated TSSs and TTSs. The RPKM were calculated based on the read counts overlapping the 5-hmC-enriched regions relative to input DNA. 5-hmC coverage profile heat maps across different genomic regions were obtained with the ngsplot tool v 2.02, considering only enriched regions^55^.

To compute 5-hmC and input DNA coverage across mouse chromosomes for each sample, the number of mapped reads in 100 kb bins was calculated and normalized for the total number of mapped reads. Boxplots of RPKM for genes enriched in 5-hmC on the TSS or in the GB were generated in R and the difference in RPKM between groups tested for significance using the Mann–Whitney test in GraphPad Prism.

For the analysis presented in Fig. 4, the four samples were clustered based on both highest stage-specific expression (measured by RNA-seq) and highest stage-specific 5-hmC coverage (calculated as number of reads falling into peaks) in genic regions.

To assess whether hydroxymethylcytosine contributed to DNA CG demethylation during cardiac development, we computed the percentage of differential CpG methylation between adult and neonatal CMCs. Genes were classified into four groups based on the difference in CpG methylation percentage (Δ≤−1, Δ≤−10, Δ≤−15 and Δ≤−20) in adult with respect to neonatal CMCs.

### RNA preparation and sequencing

Two biological replicates were profiled at each experimental point. RNA was isolated using the RNeasy Mini Kit (Qiagen) and treated with DNase according to the manufacturer's protocol. Total RNA quality was evaluated with a 2100 Agilent Bioanalyzer and the RNA 6000 Nano kit: only samples with RIN score >7 were processed further. RNA-seq libraries were created using the ScriptSeq Complete Gold—Low Input kit (Epicentre, Illumina), which is composed of two modules: the Ribo-Zero magnetic depletion protocol for ribosomal RNA removal and the ScriptSeq v2 kit to prepare the libraries. One hundred and fifty nanograms of total RNA was depleted and concentrated with RNeasy MinElute Cleanup Kit (Qiagen). Purified RNA was fragmented and first-strand complementary DNA synthesized using random hexamers with 5′-tagging sequences. After RNase digestion, a 3′-tag was introduced with random terminal tagging oligonucleotides. The obtained double-tagged strand-specific cDNA was purified using AMPure XP beads (Beckman Coulter).

Libraries were enriched and indexed by 15 cycles of PCR amplification (ABI GeneAmp PCR system 9700) using FailSafe PCR enzyme and ScriptSeq Index PCR primers. After purification with AMPure XP beads, library quality and size distribution were checked with an Agilent Bioanalyzer and DNA 1000 chip. All libraries were quantified with a Qubit 2.0 Fluorometer (Life Technologies) using dsDNA BR Assay Kit (Life Technologies). RNA-seq libraries were sequenced using TruSeq PE Cluster Kit v3 and TruSeq SBS Kit v3 (Illumina). According to index compatibility, the libraries were grouped into two pools and clustered on a TruSeq v3 flow cell at a concentration of 9 pM to obtain >50 million clusters for each sample. Paired-end sequence reads (50 bp in length) were generated on an Illumina HiSeq 2000.

### RNA-seq analysis

The correlation coefficient of the transcriptome between duplicates at the same experimental point ranged from 0.97 to 0.99, indicating strong reproducibility of the technique in our hands. Number of raw and mapped reads are reported in Supplementary Fig. 3.

The sequencing reads were processed to remove Illumina barcodes and aligned to the UCSC *Mus musculus* reference genome (build mm10), using TopHat v2.0.10 (ref. 56) with the following parameters: -r 200; --mate-std-dev 150; --library-type fr-firststrand. The reference genome was downloaded from the UCSC site and the mapped read files processed with the bamtools API 2.2.3 tool with the following parameters: -isMapped true; -isPaired true; -isProperPair. Only reads with ‘mapQ' ⩾20 were retained for further analysis. Reads were then processed by Cufflinks v2.1.1 (ref. 57) with the following parameters: -b; -u; --library-type fr-firststrand; --max-bundle-frags 10000000; --compatible-hits-norm. The RNA expression level of a gene was calculated in RPKM using the Cufflinks package. Differentially expressed genes were identified using the Cuffdiff package with the following parameters: --compatible-hits-norm; -b; u; --library-type fr-firststrand; --max-bundle-frags 10000000. Significant genes were chosen based on *q*-value (Benjamini–Hochberg correction for multiple testing) ≤0.05. Results of differential analysis are included in Supplementary Datasets 2 and 4.

Hierarchical clustering of the 10,302 differentially expressed genes between the experimental points was carried out and visualized using MeV v4.8.1 (MultiExperiment Viewer software) with complete linkage method. SOTA (Self Organizing Tree Algorithm) analysis was performed with MeV v4.8.1.

### ChIP and sequencing

Two biological replicates were profiled at each experimental point. Cells were cross-linked for 10 min at room temperature using 1% formaldehyde. Cross-linking was quenched by adding glycine to a final concentration of 0.125 M. The cells were then resuspended in lysis buffer (5 mM PIPES pH 8, 85 mM KCl, 0.5% NP40 and protease inhibitors) and incubated on ice for 15 min. Chromatin was sheared to generate 200−400 bp fragments. The efficiency of sonication was assessed with agarose gel electrophoresis. Chromatin samples were pre-cleared for 1 h with protein-G beads and then immunoprecipitated overnight at 4 °C with anti-H3 acetyl lysine 27 (Abcam, ab4729). Immunocomplexes were washed with low-salt wash buffer (0.1% SDS, 2 mM EDTA, 20 mM Tris HCl pH 8, 1% Triton X-100, 150 mM NaCl and protease inhibitors), high-salt wash buffer (0.1% SDS, 2 mM EDTA, 20 mM Tris HCl pH 8, 1% Triton X-100, 500 mM NaCl and protease inhibitors) and TE buffer. Immunocomplexes were then eluted in elution buffer (1% SDS and 100 mM NaHCO_3_) and cross-linking reverted overnight at 65 °C. Samples were treated with proteinase K, extracted with phenol/chloroform and precipitated with ethanol.

DNA was quantified using the Qubit dsDNA HS Assay Reagent Kit (Life Technologies, Q32851) on a Qubit 3.0 Fluorometer (Life Technologies, Q33216). Sequencing libraries were prepared from 5 ng of DNA using the TruSeq ChIP Library preparation kit (Illumina) according to the protocol with 18 cycles of PCR and sequenced on a NextSeq 500 (Illumina).

### ChIP-seq data processing and analysis

Previously published^23^ ChIP-seq data sets from adult and TAC CMCs were used. To profile H3K79me2, H3K4me3, H3K9ac, H3K27ac, H3K27me3, H3K9me3 and H3K9me2, mapped reads were converted from the UCSC mouse genome mm9 build to mm10 using the Batch Coordinate Conversion tool (LiftOver: https://genome.ucsc.edu/cgi-bin/hgLiftOver).

Enriched regions relative to input DNA were detected using the MACS (model-based analysis of ChIP-seq) software^58^ with the following parameters: --pvalue=1e-3 --mfold=5.30.

Intragenic levels of H3K79me2, H3K4me3, H3K9ac and H3K27ac were quantified by counting the number of reads falling into UCSC genes defined by the corresponding annotated transcription start and termination sites. The RPKMs were calculated based on the read counts overlapping the H3K79me2-, H3K4me3-, H3K9ac- and H3K27ac-enriched regions relative to input DNA. Density plots were generated in R and the Pearson's coefficient *ρ* calculated.

To profile 5-hmC distribution around the H3K79me2, H3K4me3, H3K9ac and H3K27ac MACS peaks identified in adult CMCs, we used the ‘annoatePeaks' routine in HOMER v4.5, a software suite for ChIP-seq analysis, with -size 5000 and -hist 200 as the parameters^59^.

Boxplots of RPKM for genes enriched in 5-hmC and/or the indicated histone mark were generated in R and the difference in RPKM between groups tested for significance using the Mann–Whitney test in GraphPad Prism.

### Enhancer analysis

To profile 5-hmC distribution in distal regulatory regions identified in the CMC stage^32^ and in adult and TAC CMCs^23^, enhancer regions were downloaded and converted from the mouse genome mm9 build to mm10 using the UCSC Batch Coordinate Conversion tool (LiftOver: https://genome.ucsc.edu/cgi-bin/hgLiftOver).

Enhancers were classified as 5-hmC^+^ or 5-hmC^−^ based on the presence or absence of a peak overlapping the enhancer region. Boxplots of expression for genes proximal to enhancers enriched or not in 5-hmC were generated in R and the difference in RPKM between groups tested for significance with the Mann–Whitney test in GraphPad Prism.

### Conservation of enhancers

Enhancer conservation was determined by overlapping placental mammals PhastCons elements with 5-hmC^+^ enhancer regions at each stage.

### Annotation of enriched regions

To annotate 5-hmC-enriched regions, DhMRs and ChIP regions, we used the ‘annotatePeaks' routine of HOMER v4.5.

### GO analysis

Metascape (http://metascape.org) was used to find significantly enriched (*P*-value ≤0.01) GO terms^60^. Top clusters, with their representative enriched terms (one per cluster), are given.

### Repetitive elements analysis

To study genome-wide transcriptional regulation of repetitive elements, we used the RepEnrich tool^61^. Briefly, the mouse genomic repetitive annotation required by RepEnrich was built and prepared using the RepeatMasker data set downloaded from UCSC table browsers. Raw RNA-seq reads of each sample were mapped to the mm10 genome using bowtie 4.1.2 (ref. 62) and the custom repetitive annotation prepared above with the following parameters: -m 1; --max. RepEnrich was then run with the number of total mapping reads calculated as number of reads processed minus the number of reads failing to align.

For differential enrichment analysis of repetitive elements, we used the EdgeR^63^ package in R/Bioconductor.

To correlate 5-hmC coverage on repetitive elements with the expression of the repeated DNA structures, we calculated the average number of 5-hmC reads falling into peaks overlapping all repetitive elements in each replicate, normalized for the library size (total number of mapped reads).

To compute the coverage of H3K9me3 over LINEs with or without 5-hmC in TAC CMCs, we used the ngsplot tool^55^, considering only H3K9me3 MACS-enriched regions. Boxplots of the median H3K9me3 coverage over LINEs was generated in R and the difference in values between groups tested for significance with the Mann–Whitney test in GraphPad Prism.

### Motif discovery analysis

Motif enrichment analysis within 5-hmC-associated enhancers was carried out with the ‘findMotifsGenome' routine in HOMER v4.5.

### Flow cytometric analysis

FACS analysis was carried out on six biological replicates per condition. Intracellular staining for sarcomeric α-actinin on mouse-derived CMCs was performed in the dark with the appropriate saturating concentrations of the following unconjugated antibody and relative isotype control: mouse anti-sarcomeric αactinin, diluted 1:400 (Abcam, ab9465) and normal mouse IgG (Abcam). Detection of primary antibody was performed using Alexa-647-conjugated goat anti-mouse antibody, diluted 1:500 (Molecular Probes, Invitrogen). The LIVE/DEAD Fixable Aqua Stain kit (Molecular Probes, Invitrogen) was used to exclude dead cells. Cells were fixed and permeabilized with FACS buffer (0.5% saponin and 5% fetal bovine serum). Stained cells were analysed using FACS LSRFortessa or FACSCanto II flow cytometers (BD Bioscience). Diva software (BD Pharmingen) was used for data acquisition and analysis.

### Liquid chromatography–tandem mass spectrometry quantification of 5-mC and 5-hmC

The global quantification of 5-mC, 5-hmC and 8-oxoG levels was performed according to a previously published procedure^64^. Briefly, DNA was extracted at each experimental point with QIAamp DNA mini kit (Qiagen) and dissolved in water. One microgram of DNA resulting from a pool of biological replicates in 50 μl was digested to the nucleoside level in two steps of 3 h each. Spiked-in isotope-labelled nucleosides allowed for precise quantification of levels. The quantification was performed in technical triplicates on an Agilent 1290 UHPLC system with the parameters given in Supplementary Table 1.

### TAB and regular bisulfite sequencing

TAB conversion of genomic DNA was performed with the 5-hmC TAB-Seq Kit (Wisegene) with two rounds of TET1 oxidation on four samples per condition. TAB-treated DNA or regular DNA was treated with bisulfite using the EpiTect Bisulfite kit (Qiagen), according to the manufacturer's instructions. The obtained DNA was then used for PCR amplification of the target region of interest. PCR products were purified and cloned using the Zero Blunt TOPO PCR Cloning Kit (Invitrogen). Primer sequences are listed in Supplementary Table 2.

### Biotin-based enrichment of 5-hmC

Genomic DNA was sonicated to obtain 200–500 bp fragments. Hydroxymethylated DNA fragments were enriched from 2 μg of sonicated DNA using the Hydroxymethyl Collector kit (Active Motif), according to the manufacturer's protocol. Purified DNA was evaluated by real-time PCR with SYBR green PCR master mix (Applied Biosystem). Values obtained were normalized to the input DNA. Primer sequences are listed in Supplementary Table 2.

### Gene expression analysis

RNA was extracted with RNeasy Mini columns (Qiagen) and treated with DNase (Qiagen), following manufacturer's instructions. RNA (1.2 μg) was then reverse transcribed using the Super Script VILO cDNA Syntesis Kit (Invitrogen) and cDNA was quantified by real-time qPCR with SYBR Green PCR master mix (Applied Biosystem). Expression levels of target genes were analysed with the comparative computed tomography method and normalized to 18S. Primer sequences are listed in Supplementary Table 2.

### Dot blot analysis

Genomic DNA was prepared with two-fold serial dilutions in TE buffer and denatured with 0.4 M NaOH in EDTA at 95 C for 10 min. The reaction was then blocked by addition of 2 M ice-cold ammonium acetate. Denatured DNA was spotted on a nitrocellulose membrane using a Biorad Bio-Dot apparatus and cross-linked to the membrane with ulraviolet light. The membrane was blocked with 5% milk for 2 h at room temperature and then incubated with an anti-5-hmC antibody, diluted 1:10,000 (Active Motif, 39791).

### Western blot analysis

Cell pellets were lysed in Laemmli buffer. Protein lysates were loaded on a 6% SDS–polyacrylamide gel and separated by electrophoresis. The following primary antibodies (purchased from Santa Cruz Biotechnology) were used at 4 °C overnight: anti-TET2, diluted 1:500 (sc136926) and anti-lamin B, diluted 1:500 (sc6216).

### *In vitro* silencing of *Tet2* in embryonic CMCs

Embryonic CMCs (E14.5) were plated at a density of 2.5 × 10^5^ cells per well in six-well plates and transduced with lentiviral vectors encoding a scrambled sequence as control (sh-control 5′- ATCAATCGAGCACGACTATTG -3′) or a previously validated sh-Tet2 (5′- GCTCTGAACAGTATTCAAAGC -3′)^36^. After 3 h, media was replaced and cells cultured for 72 h.

### Chromatin immunoprecipitation

ChIP analysis was performed as previously described in Papait *et al*.^23^ Briefly, 5 × 10^6^ cells were cross-linked using 1% formaldehyde. IP was performed by incubating sonicated chromatin overnight at 4 °C with specific antibodies against: MEF2C (Abcam, ab79436), 2 μg; H3K27ac (Abcam, ab4729), 3 μg; or rabbit IgG (Millipore, 12–370), 3 μg. Immunoprecipitated DNA was analysed by real-time PCR and normalized to input DNA. Primer sequences are listed in Supplementary Table 2.

### Statistical analyses

All sequencing experiments were performed on biological duplicates. Validation experiments were performed on three to four biological replicates per experimental condition. Three independent experiments were performed for *in vitro* KD of *Tet2* in embryonic CMCs. Statistical analysis was performed using Graphpad Prism 6 software or R package.

### Data availability

The complete hMeDIP and RNA-seq data sets are available from the Gene Expression Omnibus database (http://www.ncbi.nih.gov/geo/), under the accession number GSE66847.

The authors declare that all other data supporting the findings of this study are available within the article and its Supplementary Information files and from the authors upon request.

## Additional information

**How to cite this article:** Greco, C. M. *et al*. DNA hydroxymethylation controls cardiomyocyte gene expression in development and hypertrophy. *Nat. Commun.* 7:12418 doi: 10.1038/ncomms12418 (2016).

## Supplementary Material

Supplementary Information Supplementary Figures 1-11, Supplementary Tables 1-2 and Supplementary References

Supplementary Dataset 1 Lists of genomic regions enriched for 5-hmC in cardiomyocytes isolated either from E14.5 embryos, 1- or 2-day-old pups, 2-month-old adult mice or adult mice subjected to pressure overload for 1 week (TAC). The genomic coordinates are relative to the mouse genome assembly mm10. The lists are the output of HOMER.

Supplementary Dataset 2 | Transcriptional analyses of E14.5, neonatal, adult and hypertrophic cardiomyocytes. List of genes differentially expressed among the four biological conditions. (A), gene symbols; (B), genomic coordinates; (F and G), expression values, expressed as RPKM; (H), log of fold changes; (J and K), *p*- and *q*-values, respectively. The table is the result of analysis of RNA-seq data with Cuffdiff.

Supplementary Dataset 3 Differential 5-hydroxymethylated sites in embryonic, neonatal, adult and hypertrophic cardiomyocytes. Each sheet gives a list of DhMRs regions (gains and losses in 5-hmC) in the embryonic (E14.5) vs neonatal, neonatal vs adult, and normal vs hypertrophic (TAC) adult transitions. The lists are the results of analysis with MeDIPS. Genomic regions were annotated with HOMER. (A-C), genomic coordinates; (E-G), edgeR differential analysis output; (H-O), HOMER annotation output.

Supplementary Dataset 4 List of enhances in embryonic, neonatal, adult and hypertrophic cardiomyocytes. (A), chromosome; (B), start coordinates; (C), end coordinates.

Supplementary Dataset 5 Transcriptional analyses of sh-control and sh-TET2 cardiomyocytes. List of genes differentially expressed among the two biological conditions. (A), gene symbols; (B), genomic coordinates; (F and G), expression values, expressed as RPKM; (H), log of fold changes; (J and K), *p*- and *q*-values, respectively. The table is the result of analysis of RNA-seq data with Cuffdiff.

Supplementary Dataset 6 Differential 5-hydroxymethylated sites in sh-control and sh-TET2 cardiomyocytes. Each sheet gives a list of differential distribution of 5-hmC DhMRs regions (gain and loss). The lists are the results of analysis with MeDIPS. Genomic regions were annotated with HOMER. (A-C), genomic coordinates; (E-G), edgeR differential analysis output; (H-O), HOMER annotation output.
