# Supplementary material for: DNA hydroxymethylation controls cardiomyocyte gene expression in development and hypertrophy
Source: Nat Commun. 2016 Aug 4;7:12418. doi: 10.1038/ncomms12418 (PMC4976219; doi:10.1038/ncomms12418)
Supplement: Supplementary Information — Supplementary Figures 1-11, Supplementary Tables 1-2 and Supplementary References [file ncomms12418-s1.pdf]

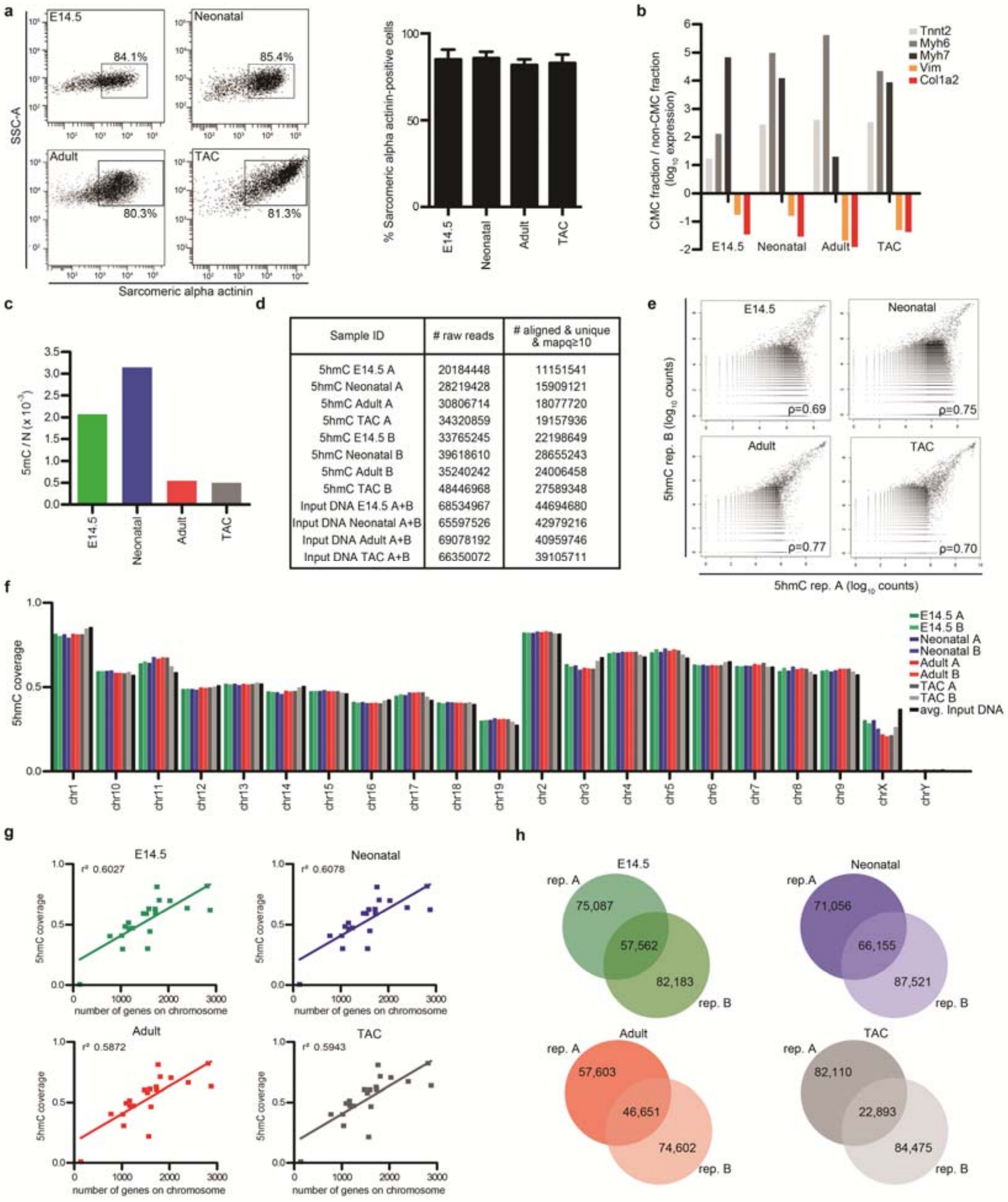

Sample ID	# raw reads	# aligned & unique & mapq≥10
5hmC E14.5 A	20184448	11151541
5hmC Neonatal A	28219428	15909121
5hmC Adult A	30806714	18077720
5hmC TAC A	34320859	19157936
5hmC E14.5 B	33765245	22198649
5hmC Neonatal B	39618610	28655243
5hmC Adult B	35240242	24006458
5hmC TAC B	48446968	27589348
Input DNA E14.5 A+B	68534967	44694680
Input DNA Neonatal A+B	65597526	42979216
Input DNA Adult A+B	69078192	40959746
Input DNA TAC A+B	66350072	39105711

Supplementary Figure 1 │ Characterization of hMeDIP sequencing results

(a) The purity of cardiomyocyte preparations was assessed with flow cytometry (FACS) for sarcomeric actinin positivity. Representative FACS plots (left) and quantification of α-actinin-positive cells (right) for embryonic (E14.5), neonatal, normal adult (Adult) and hypertrophic cardiomyocytes (TAC) are shown. Data are mean±sd (n=6).

(b) qRT-PCR analysis of cardiomyocyte and fibroblast markers in fetal and adult cardiomyocyte fractions vs the fibroblast and endothelial cell fraction.

(c) Quantification of global 5-mC in embryonic (E14.5), neonatal, normal adult (Adult) and hypertrophic cardiomyocytes (TAC), as assessed by LC–MS.

(d) Summary of raw and mapped hMeDIP sequencing reads at each of the four points studied, i.e., cardiomyocytes from embryonic (E14.5), neonatal (day 1–2), adult and transverse aortic constricted (TAC) mice, in biological replicates A and B.

(e) Pair-wise correlations ($\log_2$ hMeDIP sequencing reads) of biological replicates (A and B). For each plot, Pearson correlation coefficient ρ is shown. Genome-wide coverage profiles were computed with the MeDIPS package in R.

(f) 5-hmC and input coverage on the 19 autosomal chromosomes and the X and Y chromosomes at each of the four points studied for replicates A and B.

(g) Scatter plots showing positive correlation between the average 5-hmC coverage (replicates A and B) and the number of genes on each chromosome of the four experimental points studied.

(h) Venn diagrams showing the overlap of 5-hmC peaks between biological replicates (A and B) in embryonic, neonatal, normal adult and hypertrophic (TAC) cardiomyocytes.

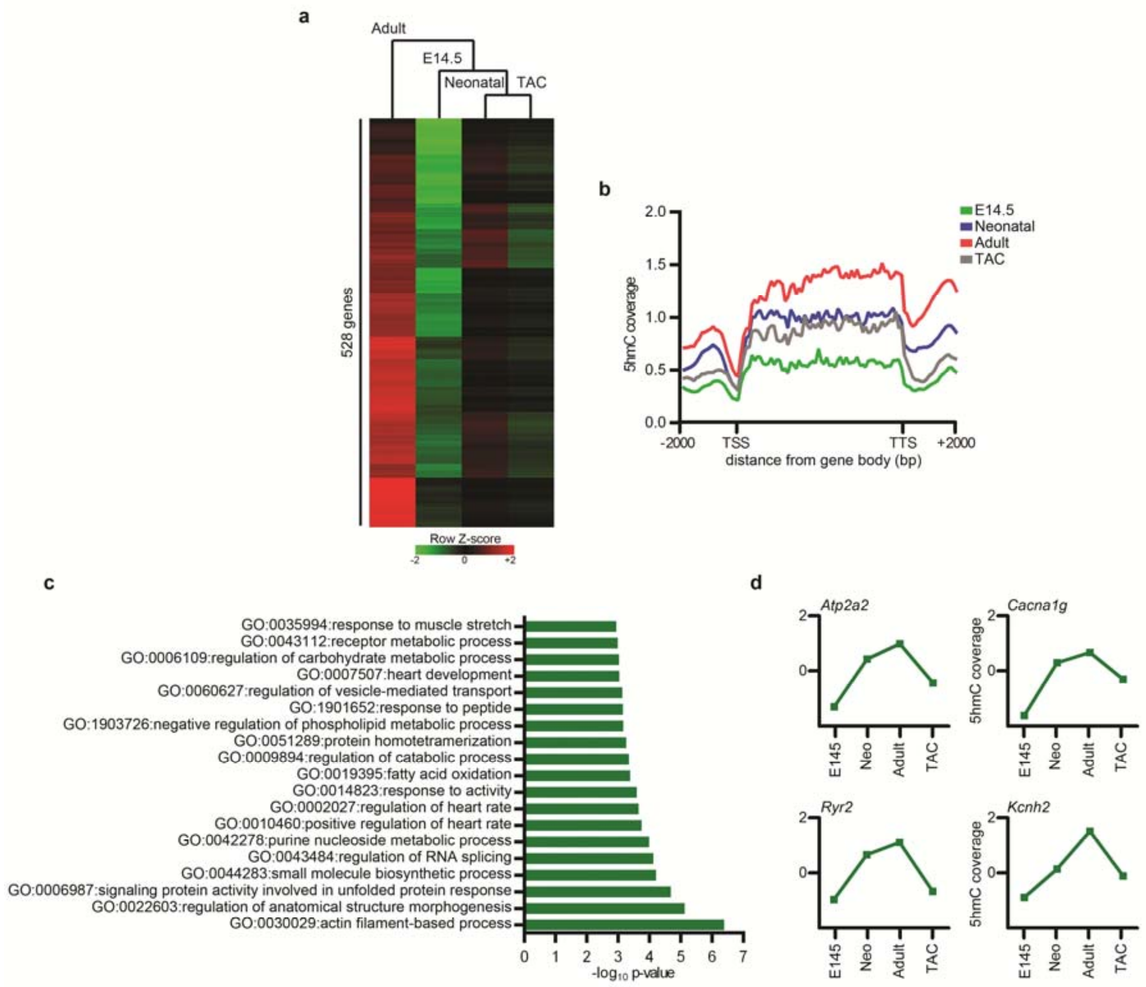

Supplementary Figure 2 │ Neonatal-like re-distribution of 5-hmC in hypertrophic cardiomyocytes

(a) Heat map of the hierarchically clustered genes undergoing a neonatal-like re-distribution of 5-hmC in TAC cardiomyocytes.

(b) Average 5-hmC coverage (RPM: reads per million mapped reads) across the gene body of the 528 genes showing neonatal-like re-distribution of 5-hmC in TAC cardiomyocytes.

(c) Enriched gene ontology terms of the 528 genes showing neonatal-like re-distribution of 5-hmC in TAC cardiomyocytes. GO analysis was performed with Metascape 1.0 (p-value ≤0.01).

(d) Examples of genes with an “immature” distribution pattern of 5-hmC deposition upon the induction of hypertrophy. Left axis represents median-normalized 5-hmC enrichment values on the gene body (exon and intron).

a

Samples	# raw reads	# aligned & paired & mapQ≥20
E14.5 A	119355608	61035372
E14.5 B	124244840	65770282
Neonatal A	117258274	62871492
Neonatal B	122387254	66057960
Adult A	113487562	53582036
Adult B	105547836	51890850
TAC A	102371446	51911496
TAC B	110992160	55348842

b

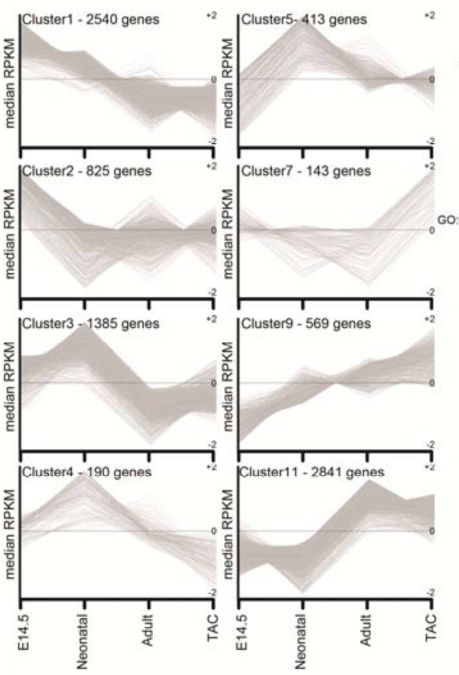

c

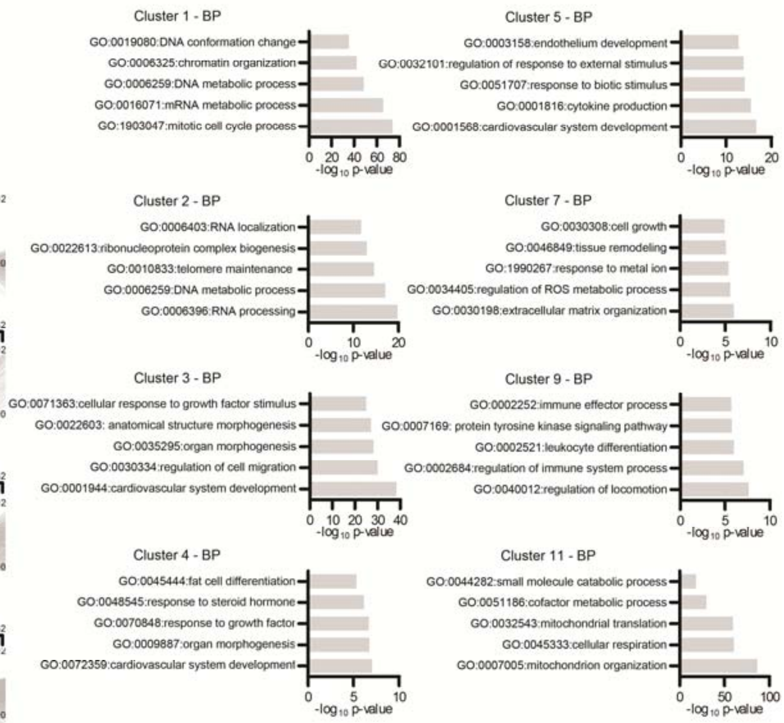

Supplementary Figure 3 │ Characterization of RNA sequencing results

(a) Summary of raw and mapped RNA sequencing reads in 2 replicates (A and B) per studied point.
(b) SOTA clustering analysis of the 10,302 differentially expressed genes. Clusters not given in Fig. 2 are shown. Y axis represent median-normalized RPKM values (-2; +2).
(c) Enriched gene ontology terms of the relative SOTA cluster. GO analysis was performed with Metascape 1.0 (p-value ≤0.01).

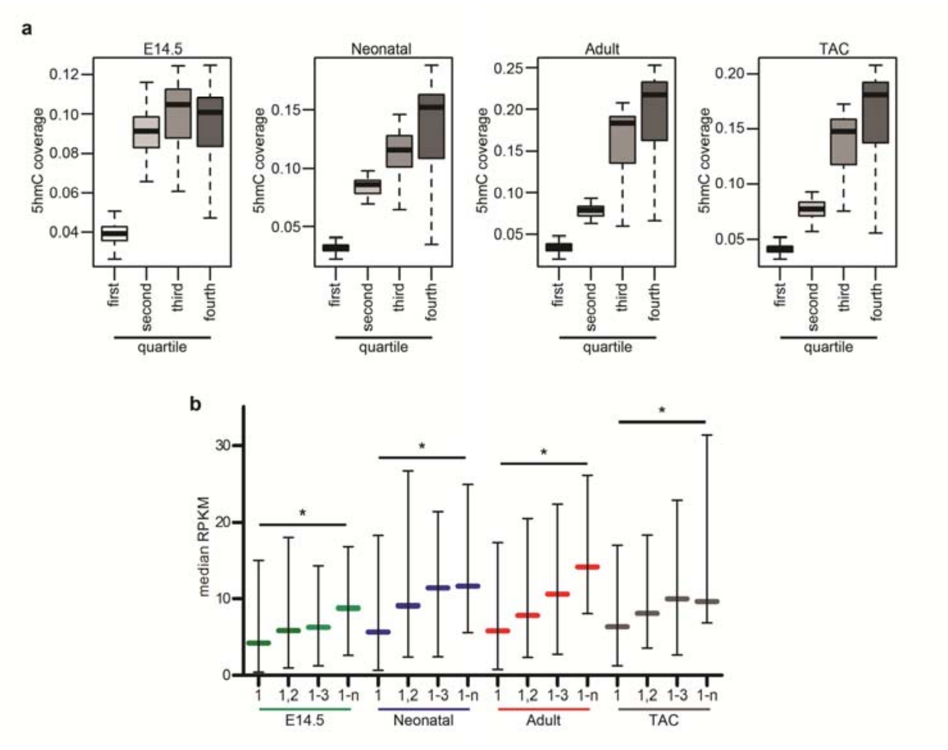

Supplementary Figure 4 │ Correlation of 5-hydroxymethylcytosine with gene expression during cardiomyocyte development and hypertrophy

(a) Average 5-hmC coverage (RPM) over the two replicates from the TSS to the TTS on genes clustered into quartiles of expression. Boxplots of median, with whiskers extending to ±1.5 of the interquartile range.

(b) Plot of median RPKM values of genes harbouring 5-hmC only at the first intron (1), at the first two introns (1, 2), at the first three introns (1–3) and at more than three introns (1–n). *, *p*-value ≤0.01 (Mann-Whitney test).

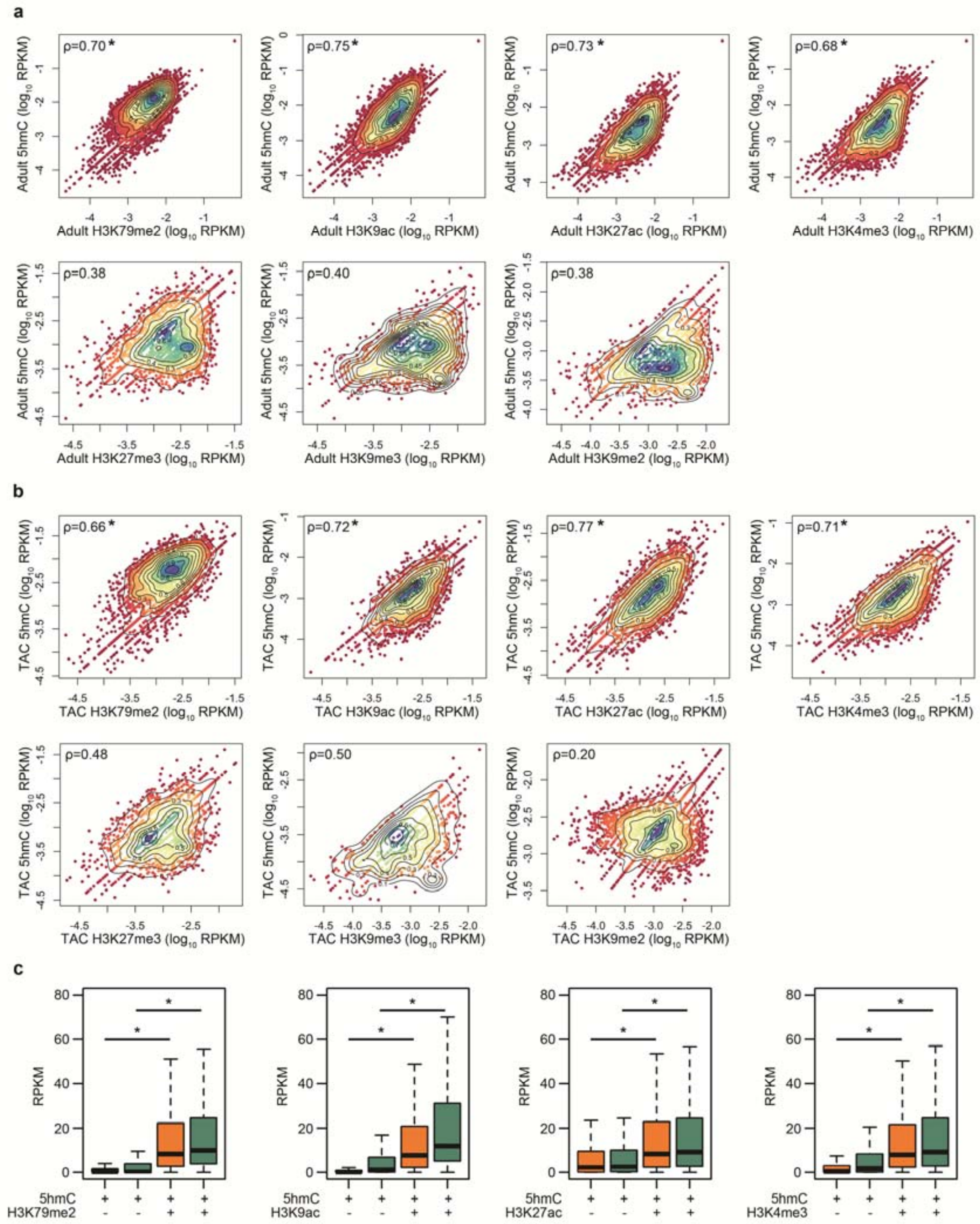

Supplementary Figure 5 | Correlation of 5-hydroxymethylcytosine with activating histone marks in adult cardiomyocytes

(a,b) Density plots showing correlation of the gene-body 5-hmC levels in normal (a) and hypertrophic (b) adult cardiomyocytes with gene-body levels of activating (H3K79me2, H3K9ac, H3K27ac and H3K4me3) and repressing (H3K27me3, H3K9me3 and H3K9me2) histone marks[1]. The Pearson correlation coefficient ρ is given for each plot. *, *p*-value $<2\times10^{-16}$ (two-tailed). Intragenic levels of 5-hmC, H3K79me2, H3K4me3, H3K9ac and H3K27ac were quantified by counting the number of reads falling on genes from the TSS to the TTS.

(c) RNA sequencing expression values in normal adult (orange bars) and hypertrophic (TAC, green bars) cardiomyocytes for genes positive exclusively for 5-hmC on the gene body and of genes positive for 5-hmC and the indicated histone mark on the gene body. Boxplots give the median (bold line), with whiskers extending to ±1.5 of the interquartile range. *, *p*-value ≤0.01 (Mann-Whitney test).

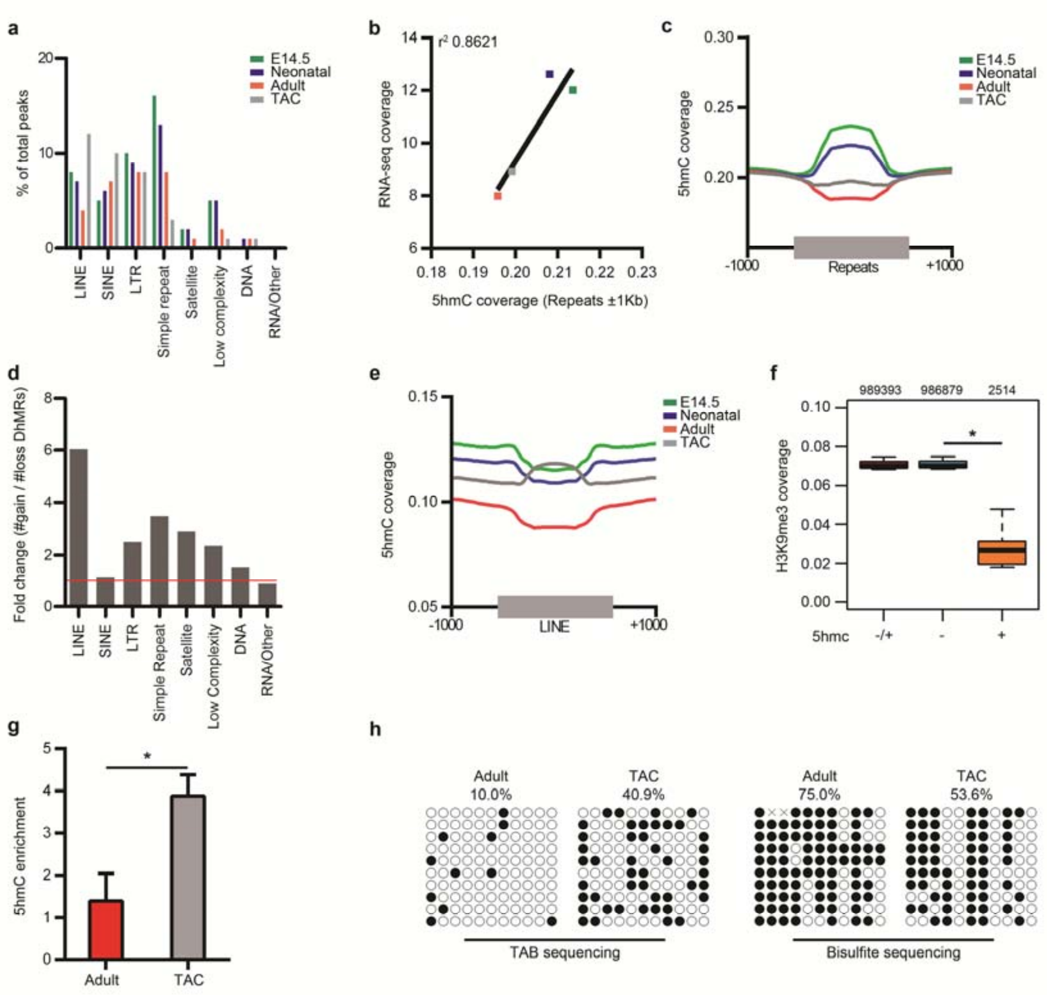

Supplementary Figure 6 | Association of 5-hydroxymethylcytosine with repetitive elements of the genome

(a) Analysis of repeat masker elements (UCSC version mm10) associated with 5-hmC peaks (assessed by HOMER) at the four studied points.
(b) Positive correlation between 5-hmC coverage and expression of repeat elements in cardiomyocytes.
(c) 5-hmC coverage (RPM) across the pooled repeat elements.
(d) Fold change in the number of differentially hydroxymethylated regions (DhMRs) found in the normal-to-hypertrophic transition in adult cardiomyocytes, for each repeat masker class.
(e) 5-hmC coverage (RPM) across LINEs at the four studied points.
(f) Coverage of H3K9me3 on LINEs in hypertrophic cardiomyocytes. The boxplot gives the median (bold line), with whiskers extending to ±1.5 of the interquartile range. LINEs enriched (+) or not (-) in 5-hmC. *, *p*-value ≤0.01 (Mann-Whitney test).
(g) Quantitative PCR analysis of 5-hmC enrichment on LINE-1. *, *p*-value ≤0.01 (unpaired Student's t-test). Data are presented as mean±sd (n=3).
(h) Detection of 5-hmC (TAB sequencing) and 5-mC by single-base resolution analysis (regular bisulfite sequencing) at LINE-1 in adult and hypertrophic (TAC) cardiomyocytes. Black circles indicate methylated or hydroxymethylated CpGs; open circles indicate unmethylated CpGs; x indicates undetermined.

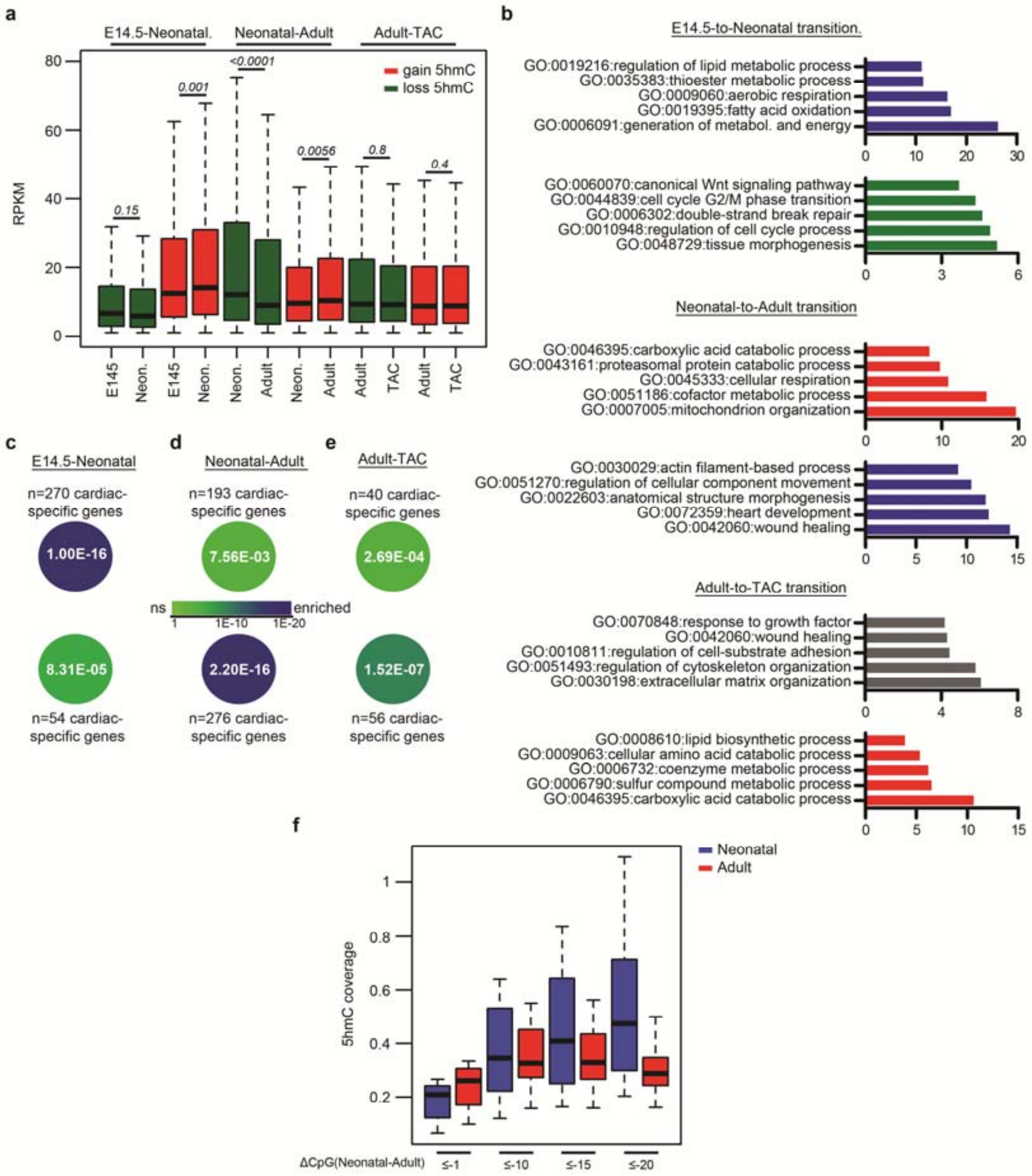

Supplementary Figure 7 | Analysis of differentially hydroxymethylated regions (DhMRs)

(a) RNA sequencing expression values in embryonic (E14.5), neonatal (Neon.), adult and hypertrophic (TAC) cardiomyocytes for genes exclusively losing 5-hmC (green boxes) and genes exclusively gaining 5-hmC (red boxes) in the three transitions. Boxes: 1-4, embryonic-to-neonatal transition; 5-8, neonatal-to-adult transition; 9-12, normal adult-to-hypertrophic transition. Boxplots give the median (bold line), with whiskers extending to ±1.5 of the interquartile. *p*-values were calculated with the Mann-Whitney test.

(b) Enriched gene ontology terms related to significantly up-regulated (blue) and down-regulated (green) genes with DhMRs during the embryonic-to-neonatal transition (top 2 graphs); significantly up-regulated (red) and down-regulated (blue) genes with DhMRs during the neonatal-to-adult transition (middle two graphs); and significantly up-regulated (grey) and down-regulated (red) genes with DhMRs during the adult-to-TAC transition (bottom two graphs). GO analysis was performed with Metascape 1.0 (*p*-value ≤0.01).

(c) Number of cardiac-specific up- (top) and down- (bottom) regulated genes among the total number either gaining or losing 5-hmC in the embryonic-to-neonatal transition (see Fig. 4e in main text). Enrichment for genes with MGI cardiovascular expression was calculated using Fisher's Exact test (ns: not significant).

(d) Number of cardiac-specific up- (top) and down- (bottom) regulated genes among the total number either gaining or losing 5-hmC in the neonatal-to-adult transition (see Fig. 4f in main text). Enrichment for genes with MGI cardiovascular expression was calculated using Fisher's Exact test.

(e) Number of cardiac-specific up- (top) and down- (bottom) regulated genes among the total number either gaining or losing 5-hmC during the induction of hypertrophy (see Fig. 4g in main text). Enrichment for genes with MGI cardiovascular expression was calculated using Fisher's Exact test.

(f) 5-hmC coverage (RPM) at developmentally dynamic demethylated CG regions undergoing differential CpG methylation percentages of ≤−1, ≤−10, ≤−15 and ≤−20 in the neonatal-to-adult transition.

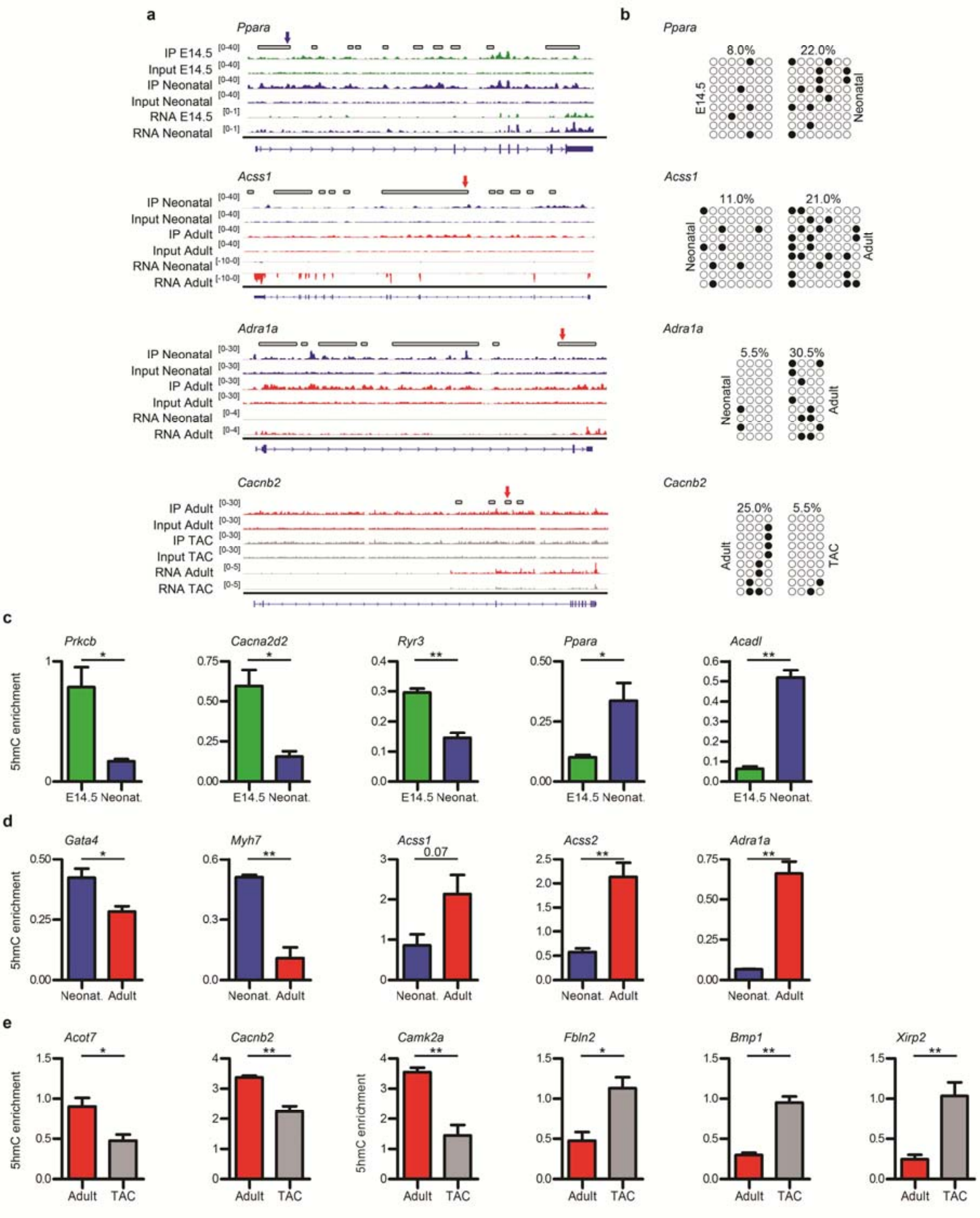

Supplementary Figure 8 | Validation of differentially hydroxymethylated regions (DhMRs)

(a) IGV profiles of 5-hmC MeDIP-seq and RNA-seq profiles of representative genes (*Ppara*, *Acss1*, *Adra1a* and *Cacnb2*). Exact locations of primers used to validate DhMRs regions are shown above the IGV profile (blue arrow: neonatal-to-adult transition; red arrow: adult-to-TAC transition). Primer sequences are listed in Supplementary Table S2.

(b) Validation of 5-hmC by single-base resolution analysis (TAB sequencing). Black circles indicate hydroxymethylated CpGs; open circles indicate unmethylated CpGs. x indicates CpGs with undefined methylation status. Exact location of primers used to validate selected regions are listed in Supplementary Table S2.

(c) Quantitative PCR validation of differentially hydroxymethylated regions in the embryonic-to-neonatal transition .

(d) Quantitative PCR validation of differentially hydroxymethylated regions in the neonatal-to-adult transition.

(e) Quantitative PCR validation of differentially hydroxymethylated regions between normal adult and hypertrophic (TAC) cardiomyocytes. Data are presented as mean±sd (n=3). *, p-value ≤0.05; **, p-value ≤0.01 (unpaired Student's t-test).

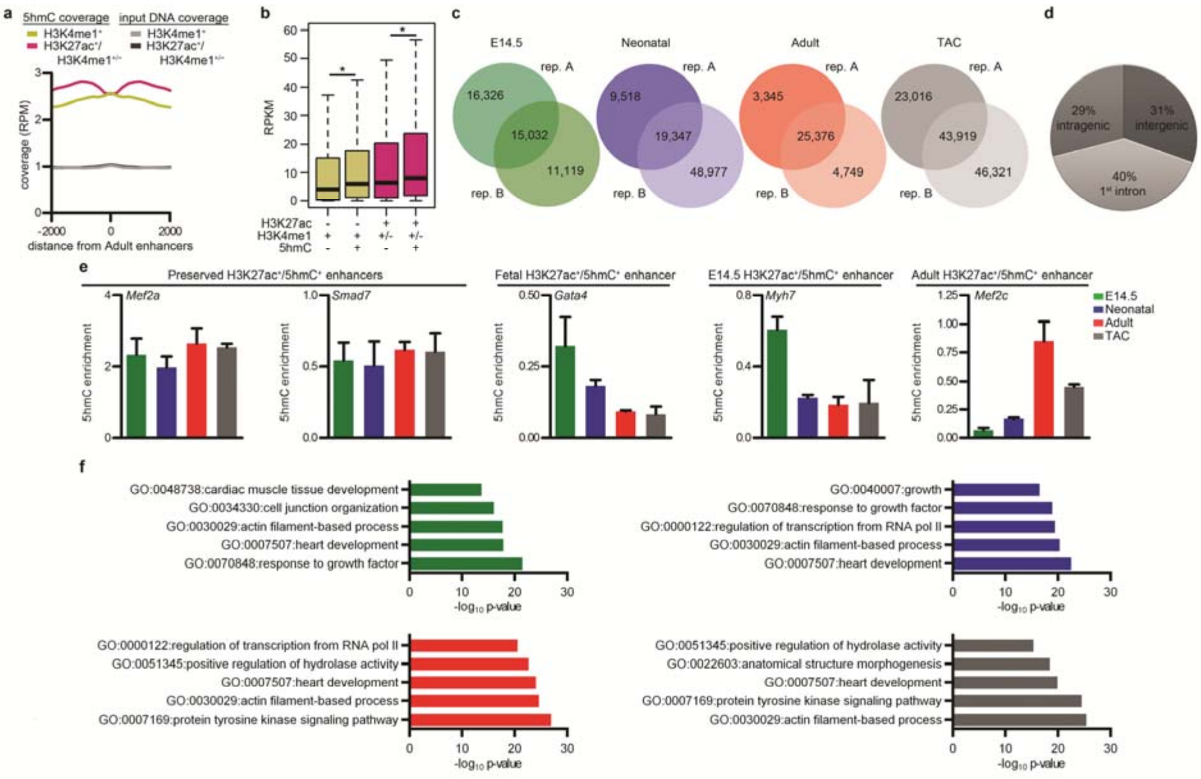

Supplementary Figure 9 | Association of 5-hydroxymethylcytosine with enhancers

(a) Profiles of 5-hmC and input DNA coverage across poised ($H3K4me1^+$) and active ($H3K27ac^+/H3K4me1^{+/-}$) enhancers identified in cardiomyocytes differentiated *in vitro* from embryonic stem cells, as defined in Wamstad et al.[2] RPM, read counts per million mapped reads. Distance from adult enhancers given in bp.

(b) Boxplots (whiskers extending to ± 1.5 of the interquartile range) of adult RNA sequencing expression values (RPKM) for genes near poised/active enhancers enriched or not for 5-hmC. *, *p*-value ≤0.01 (Mann-Whitney test).

(c) Venn diagram showing the overlap of H3K27ac peaks between biological replicates (A, B) in embryonic, neonatal, normal adult and hypertrophic cardiomyocytes.

(d) Pie chart illustrating the distribution of all 43,005 $H3K27ac^+$ enhancers across the genome.

(e) Quantitative PCR validation of $H3K27ac^+$/5-hmC^+ enhancers found near a cardiac-specific gene at all stages (Preserved), at embryonic and neonatal stages (Fetal), at only the embryonic stage (E14.5) and at only the adult stage (Adult). Data presented as mean±sd (n=3).

(f) Enriched gene ontology terms of genes near $H3K27ac^+$/5-hmC^+ enhancers in embryonic (green), neonatal (blue), adult (red) and hypertrophic (grey) cardiomyocytes. GO analysis was performed with Metascape 1.0 (*p*-value ≤0.01).

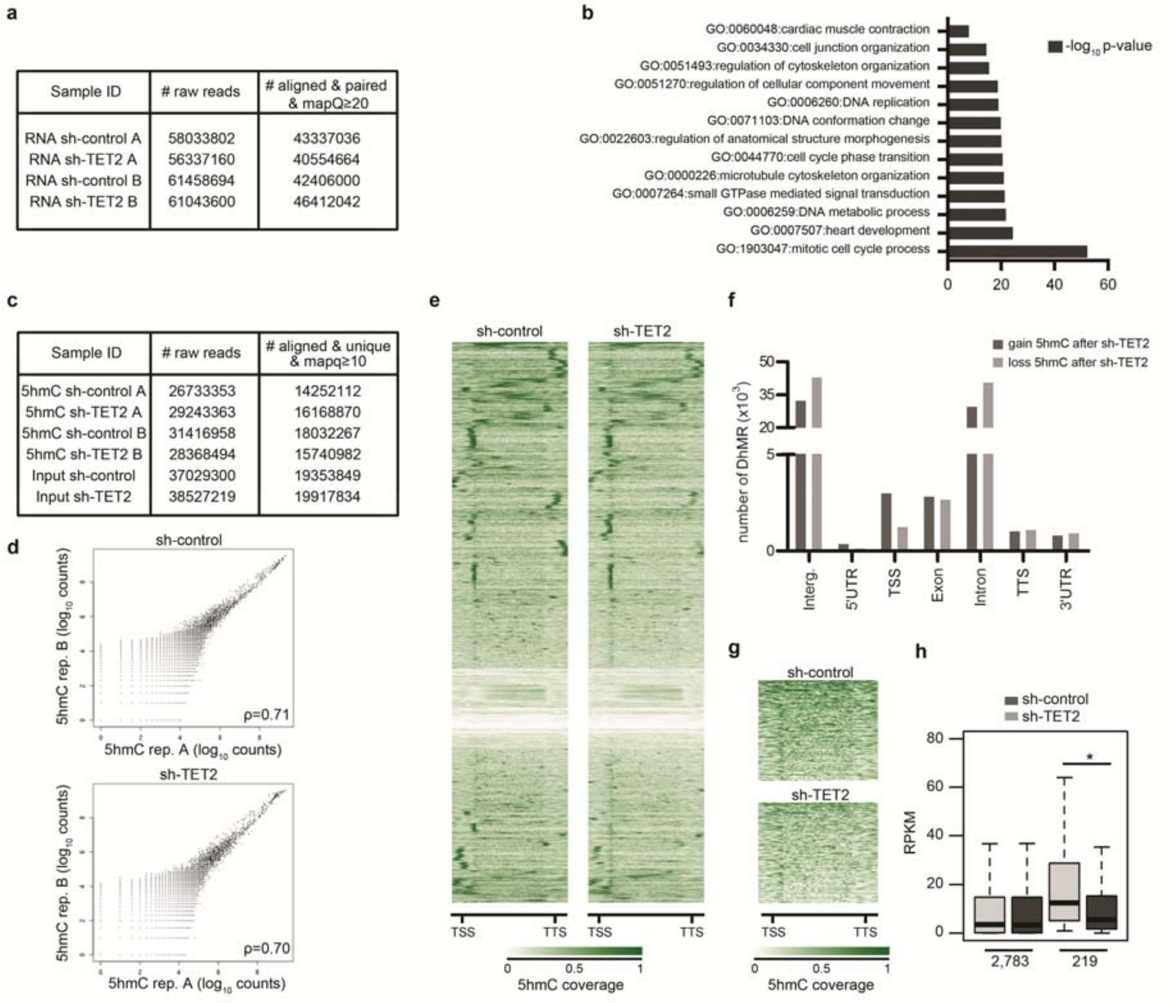

Supplementary Figure 10 | Sequencing results of *Tet2* knockdown in embryonic cardiomyocytes

(a) Summary of raw and mapped RNA sequencing reads in 2 samples (A and B) at each studied point.

(b) Enriched gene ontology terms of genes down-regulated after *Tet2* KD. GO analysis was performed with Metascape 1.0 (*p*-value ≤0.01).

(c) Summary of raw and mapped hMeDIP sequencing reads for each replicate.

(d) Pair-wise correlation of biological replicates A and B (log_2 hMeDIP sequencing reads). For each plot, Pearson correlation coefficient ρ is shown. Genome-wide coverage profiles were computed with MeDIPS in R package.

(e) Heat map of 5-hmC densities on gene bodies of all reference genes (UCSC version mm10) and flanking regions (±2Kb). There is no clear difference in the global profile of the two samples.

(f) Association of all identified DhMRs (between sh-control and sh-TET2 cardiomyocytes) with different genomic elements.

(g) Heat map of 5-hmC densities on gene bodies of the 219 genes undergoing both loss of 5-hmC on the gene body (measured by hMeDIP) and down-regulation of expression (measured by RNA-seq) after sh-TET2 KD.

(h) Boxplots (median RNA expression with whiskers extending to ±1.5 of the interquartile range) of RNA sequencing expression values (RPKM) for all genes losing 5-hmC on the gene body (n=2,783) and for the 219 genes losing 5-hmC on the gene body and becoming down-regulated after *Tet2* KD. *, *p*-value ≤0.01 (Mann-Whitney test).

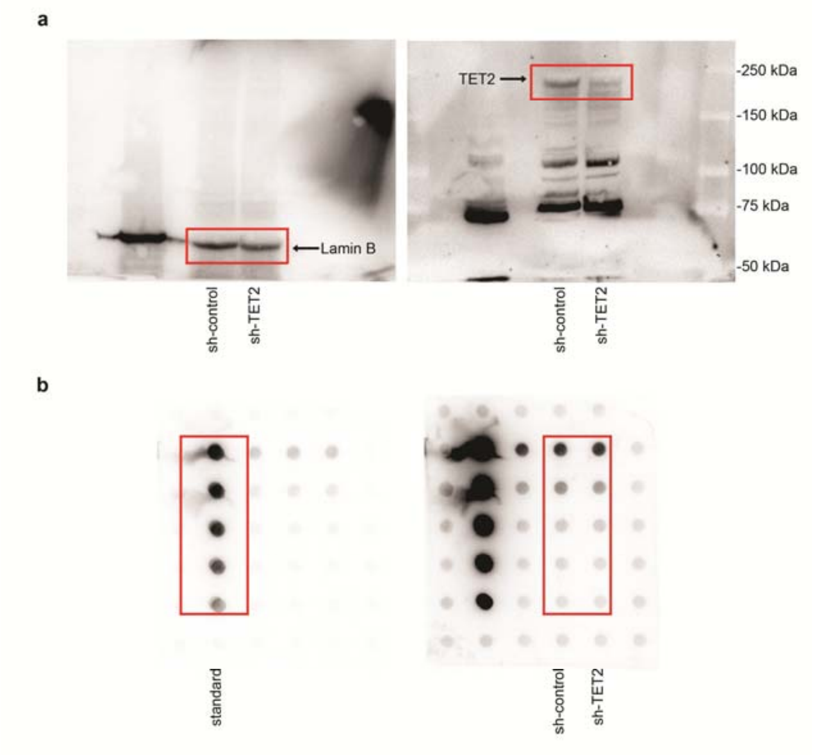

Supplementary Figure 11 | Full Western and dot blots

(a) Full Western blots for lamina B and TET2, related to Figure 6b.
(b) Full dot blots, related to Figure 6c.

Supplementary Table 1 │Parameters used for LC-MS/MS quantification of 5-mC and 5-hmC

Compound	Precursor Ion (*m/z*)	MS1 Resolution	Product Ion (*m/z*)	MS2 Resolution	Dwell time [ms]	CE (V)	CAV (V)	Polarity
Time segment 1.5–4.0 min								
[$^{15}N_2$,D_2]-hmC	262.12	Wide	146.07	Wide	40	8	1	+
hmC	258.11	Wide	142.06	Wide	40	8	1	+
[D_3]-mC	245.13	Wide	129.09	Wide	30	60	1	+
mC	242.11	Wide	126.07	Wide	30	60	1	+
Time segment 6.0–9.0 min								
[$^{15}N_5$]-8-oxo-G	289.08	Wide	173.04	Wide	120	9	7	+
8-oxo-G	284.1	Wide	168.05	Wide	120	9	7	+

Supplementary Table 2 │ Primer List

Biotin-based enrichment of 5-hmC

Refers to Supplementary Figure 8

**Comparison**	**Gene**	**Forward Primer (5' → 3')**	**Reverse Primer (5' → 3')**
E14.5 vs Neonatal	*Cacna2d2*	caggatgacagtctgcaaaga	ttcagctcgcagaaatttga
E14.5 vs Neonatal	*Prkcb*	ggaagccagaaagaagctca	gggagtcacgagttcagca
E14.5 vs Neonatal	*Ryr3*	cttctgttcagcccatttcc	ggtgactgtgcgttaggactt
Neonatal vs E14.5	*Acadl*	ctcccaccacagcattttg	tccaccttaccgatttccaa
Neonatal vs E14.5	*Ppara*	ggaaggagagagtgtgctggt	ggctcagatccaatcacagag
Neonatal vs Adult	*Gata4*	caagccctctcctgagaaca	gcgaacaaaagcctactgct
Neonatal vs Adult	*Myh7*	gatttgggggaaggtgtctt	tgcactgctcaagcctaaag
Adult vs Neonatal	*Acss1*	gccatgtcaggtcacatcc	tgcgggggtataattttgg
Adult vs Neonatal	*Acss2*	ttgctgtcacatagaaaacctaaga	gagcagttggcgctcttaac
Adult vs Neonatal	*Adra1a*	tccttaccaacatgccaaca	aatagcctaaccattgggaaca
Adult vs TAC	*Acot7*	catggtcctagttgctggttc	gtcaggtattcgagcgatcc
Adult vs TAC	*Cacnb2*	aacaaatgatacgtgggagtca	gcctgagcttgttcataccc
Adult vs TAC	*Camk2a*	caagaaaaagggcaagtcaga	ctcagcatcacccagccta
TAC vs Adult	*Fbln2*	tgagtgccttcctacagaagc	gctagagccacagcagaaca
TAC vs Adult	*Xirp2*	catctctcaatctcaggctcttt	catcaggtcatgctttgtgg
TAC vs Adult	*Bmp1*	tgtatcaccctcttgcgtttt	tcccgtgtggatacttaggg

Refers to Supplementary Figure 6

**Comparison**	**Gene**	**Forward Primer (5' → 3')**	**Reverse Primer (5' → 3')**
TAC vs Adult	*LINE-1*	cactcccaccccacctagt	taactctttagcagtgctctcctgt

Refers to Figure 6

**Comparison**	**Gene**	**Forward Primer (5' → 3')**	**Reverse Primer (5' → 3')**
sh-control vs sh-TET2	*Myl4*	ctccattccccctctcaact	acgggaggtagagagtgcag
sh-control vs sh-TET2	*Myh7*	tggaggctgaagccttactt	ggctcgagtctgaaatctgg

Refers to Supplementary Figure 9

**Enhancer proximal to**	**Forward Primer (5' → 3')**	**Reverse Primer (5' → 3')**
Mef2a	ggaggagacggaatcagaaa	gcactaggttcttggcttgg
Smad7	cagagcaggaccccagatt	acttgagcagagagatgctgtg
Gata4	ggaagctcgaagaccaagtg	cctgctacagtggtgggaat
Myh7	gggactgggacataggatca	tgccctctcctctccttgt
Mef2c	ccgctcttttacacgacctatc	gactggagacgaatgggaaa

Refers to Figure 7

**Enhancer proximal to**	**Forward Primer (5' → 3')**	**Reverse Primer (5' → 3')**
Myh7	gggactgggacataggatca	tgccctctcctctccttgt
Myl3	gacattactgtccccacactgtc	ttgggagcctaattcagcat
Hif1a	ttggatctggggaagaactg	atgtgaccatgggagctggg
Gata4	ggaagctcgaagaccaagtg	cctgctacagtggtgggaat

TAB seq

Refers to Figure 4

**Comparison**	**Gene**	**Forward Primer (5' → 3')**	**Reverse Primer (5' → 3')**
E14.5 vs Neonatal	*Myh7*	ttgagttgggttggataaaggt	ccaactaaaaaaatccctaaaacaa
Neonatal vs Adult	*Myh7*	tgagtttaggaatgtatagggtatttttt	cctcaaaacactcaaatccaacta

Refers to Supplementary Figure 8

**Comparison**	**Gene**	**Forward Primer (5' → 3')**	**Reverse Primer (5' → 3')**
Neonatal vs E14.5	*Ppara*	aatggttttaggaagaaaatagataa	attcttcaaatacacccatacacac
Adult vs Neonatal	*Acss1*	gtattttttgtttttgggattgttt	ccaactctttaaacttccaacctac
Adult vs Neonatal	*Adra1a*	aaatgagaagttgggtgtttttaat	caaatttttaaattctttttcaacc
Adult vs TAC	*Cacnb2*	aatttttaatttttgtgtatgtaatg	cctcaaataatacttctaataaaaaac

Refers to Supplementary Figure 6

Comparison	Gene	Forward Primer (5' → 3')	Reverse Primer (5' → 3')
TAC vs Adult	*LINE-1*	taggaaattagtttgaataggtgagag	tcaaacactatattactttaacaattc

qRT-PCR expression

Refers to Supplementary Figure 1

Gene	Forward Primer (5' → 3')	Reverse Primer (5' → 3')
Tcf21	tgcgccagcagtatgaaa	gcctcagagaggtcagcaaa
Col1a2	cattcacccagtcaacctga	ccacttccttcaggtcattctc
Vim	tgaagtgggtcttccaggtc	acaccagggagtccagtagc

ChIP

Refers to Figure 5

Gene	Forward Primer (5' → 3')	Reverse Primer (5' → 3')
Myh7	gaccacacagaaagctcctga	tcctgagggccacagttta
Myh7	ctgctgtgagagaaggcaga	ctccctaacacccttgtgct

Refers to Figure 7

Gene	Forward Primer (5' → 3')	Reverse Primer (5' → 3')
Myh7	gggactgggacataggatca	tgccctctcctctccttgt
Myl3	gacattactgtccccacactgtc	ttgggagcctaattcagcat
Hif1a	ttggatctggggaagaactg	atgtgaccatgggagctggg
Gata4	ggaagctcgaagaccaagtg	cctgctacagtggtgggaat

Supplementary References

1. Papait, R., et al. Genome-wide analysis of histone marks identifying an epigenetic signature of promoters and enhancers underlying cardiac hypertrophy. Proc. Natl. Acad. Sci. U.S.A. 110, 20164-20169 (2013).
2. Wamstad, J.A., et al. Dynamic and coordinated epigenetic regulation of developmental transitions in the cardiac lineage. Cell 151, 206-220 (2012).
